# Inflammation-induced TRIM21 represses hepatic steatosis by promoting the ubiquitination of lipogenic regulators

**DOI:** 10.1172/jci.insight.164694

**Published:** 2023-11-08

**Authors:** Kostas C. Nikolaou, Svenja Godbersen, Muthiah Manoharan, Stefan Wieland, Markus H. Heim, Markus Stoffel

**Affiliations:** 1 Institute of Molecular Health Sciences, ETH Zurich, Zürich, Switzerland.; 2 Alnylam Pharmaceuticals, Cambridge, Massachusetts, USA.; 3 Department of Biomedicine, University of Basel, Basel, Switzerland.; 4 Clarunis, University Center for Gastrointestinal and Liver Diseases, Basel, Switzerland.; 5 Medical Faculty, University of Zürich, Zürich, Switzerland.

**Keywords:** Hepatology, Diabetes

## Abstract

Nonalcoholic steatohepatitis (NASH) is a leading cause for chronic liver diseases. Current therapeutic options are limited due to an incomplete mechanistic understanding of how steatosis transitions to NASH. Here we show that the TRIM21 E3 ubiquitin ligase is induced by the synergistic actions of proinflammatory TNF-α and fatty acids in livers of humans and mice with NASH. TRIM21 ubiquitinates and degrades ChREBP, SREBP1, ACC1, and FASN, key regulators of de novo lipogenesis, and A1CF, an alternative splicing regulator of the high-activity ketohexokinase-C (KHK-C) isoform and rate-limiting enzyme of fructose metabolism. TRIM21-mediated degradation of these lipogenic activators improved steatosis and hyperglycemia as well as fructose and glucose tolerance. Our study identifies TRIM21 as a negative regulator of liver steatosis in NASH and provides mechanistic insights into an immunometabolic crosstalk that limits fatty acid synthesis and fructose metabolism during metabolic stress. Thus, enhancing this natural counteracting force of steatosis through inhibition of key lipogenic activators via TRIM21-mediated ubiquitination may provide a therapeutic opportunity to treat NASH.

## Graphical abstract

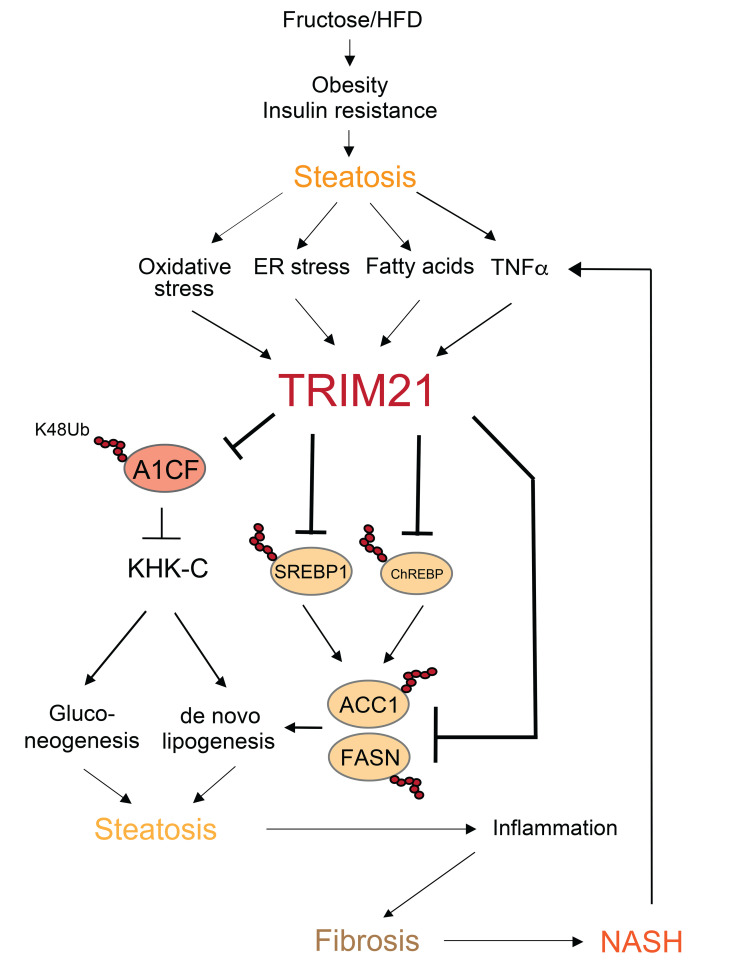

## Introduction

Nonalcoholic fatty liver disease (NAFLD) is associated with the metabolic syndrome that is characterized by obesity, type 2 diabetes mellitus, dyslipidemia, and hypertension (1–3). It is the most common cause of liver dysfunction worldwide, and recent epidemiological evidence implicates it as the leading etiology underlying chronic liver diseases (4). The incidence of NAFLD has increased rapidly, reflecting the rise of the global obesity epidemic, with estimates of 25% prevalence in the overall population (5, 6). Approximately 7%–30% of individuals with NAFLD are estimated to develop nonalcoholic steatohepatitis (NASH), a more advanced liver disease with pathophysiological characteristics of excessive lipid accumulation, inflammation, injury, and fibrosis, which puts subjects at high risk for progressing to cirrhosis and hepatocellular carcinoma (4, 7–9). Pharmacological treatments of NASH are limited, and few drugs are currently under clinical evaluation (10, 11). Thus, further studies are needed to identify key molecular regulators of NASH progression and to develop more effective therapies.

The pathogenesis of NAFLD and the progression from benign hepatic steatosis to NASH is complex and not entirely understood. Sedentary lifestyle, high-caloric intake, hepatic insulin resistance (IR), and genetic predisposition have been recognized for some time as factors accelerating metabolic-related chronic liver pathology (1–3, 12–14). Accumulating evidence points toward hepatic de novo lipogenesis (DNL) as a key mediator in the development of NAFLD (15). Exponential fructose consumption is also recognized as an important driving force for the development of NAFLD, since fructose overconsumption in mice and humans triggers lipogenesis by activating the transcription factors SREBP1c and ChREBP and augmenting the expression of lipogenic enzymes, influencing hepatic steatosis, IR, dyslipoproteinemia, and adiposity (16–26). Fructose is metabolized by ketohexokinase (KHK), the rate-limiting enzyme of fructolysis in both the liver and intestine (26–28); however, mouse genetic studies have suggested that only hepatic fructose catabolism drives steatosis (29).

We have recently identified a major role for the RNA-binding protein A1CF as a hepatocyte-specific alternative splicing factor that regulates the processing of liver-enriched mRNA isoforms, including those of *Khk-C* and glycerol kinase (*Gk*), 2 key metabolic enzymes linked to hepatic lipogenesis and gluconeogenesis. Liver-specific deletion of A1CF in mice resulted in markedly reduced expression of these enzymes and, consequently, prevented fructose-induced hyperglycemia, hyperinsulinemia, steatosis, and adiposity (30), thereby phenocopying KHK deficiency in mice (31).

Liver steatosis is primarily driven by the transcription factors SREBP1c and ChREBP, which exert synergistic effects on lipogenic gene expression, thereby regulating hepatic DNL and contributing to steatosis in individuals with NAFLD. Hepatic SREBP1c and ChREBP levels are overinduced in livers of obese animals (32–34), and their expression and activity is controlled by nutritional and hormonal factors, by insulin and glucose, respectively (35). While many studies have investigated the molecular mechanisms that result in activation of SREBP1c and ChREBP and their target networks as well as their contribution to steatosis development, our knowledge of how these pathways are turned off is scarce. The ubiquitin ligases FBW7 and SMURF2 have been shown to degrade SREBPs and ChREBP via the proteasome pathway (36, 37), thereby influencing lipid metabolism and aerobic glycolysis, respectively. However, these degradation pathways have only been studied in mitotic cells of cancer tissues and cell lines, and their role in livers exposed to metabolic stress is unclear. Furthermore, recent in vivo studies of members of TRIM family of E3-ubiquitin ligases have indicated their major role in the control of metabolic homeostasis and development of chronic liver disease (38–40).

Here, we report that, in inflamed fatty livers of mice and humans E3 ubiquitin ligase, TRIM21 is induced by the synergistic action of TNF-α and fatty acids, the principal drivers of NASH. We demonstrate that, during NAFLD progression, TRIM21 suppresses hepatic lipogenesis and fructolysis on a transcriptional, RNA splicing and enzymatic level by polyubiquitination and degradation of SREBP1, ChREBP, A1CF, ACC1, and FASN. Our results reveal a protective mechanism that counteracts hepatic steatosis progression under metabolic stress and TNF-α proinflammatory signals.

## Results

### TRIM21 interacts, ubiquitinates, and degrades A1CF in hepatocytes.

**Figure 1:**
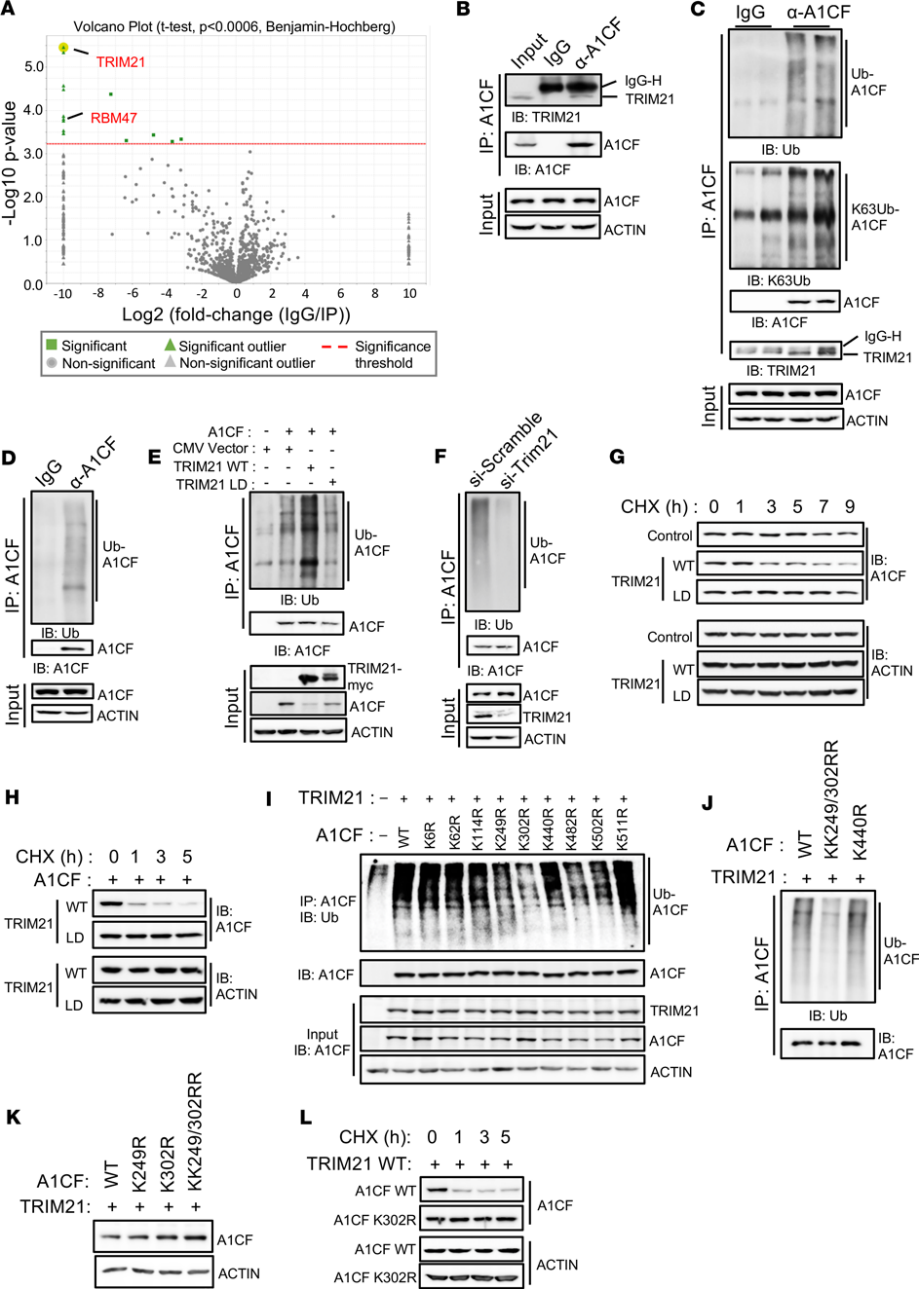
TRIM21 interacts, ubiquitinates, and degrades A1CF in hepatocytes. (**A**) Volcano plot of proteomics data, depicting protein data *P* values versus fold change (FC). Normalized total ion current (TIC) was used as a quantitative method to plot the volcano. Proteomics data were produced from 3 liver replicates, 1 mouse/replicate. All data points above threshold (red line) are calculated with *t* test significance *P* < 0.05 and Benjamini-Hochberg multiple-test correction. All significant proteins associated with these points are listed in Supplemental Table 1. Highlighted are TRIM21 and RBM47, a known A1CF interactor. (**B**) Coimmunoprecipitation (Co-IP) experiments to validate the endogenous A1CF and TRIM21 interaction in mouse livers. (**C** and **D**) Ubiquitination (Ub) assay determining either K63-linked Ub-specific or total Ub of endogenous A1CF in mouse liver (**C**) or HepG2 cells (**D**); ubiquitination assay shown in **D** was performed in HepG2 cells. (**E**) Total A1CF ubiquitination levels in Hepa 1–6 cells cotransfected with A1CF and either TRIM21 WT or its ligase-dead (LD) form after MG132 (0.5 μM) treatment for 6 hours. Input levels indicate A1CF and TRIM21 expression. (**F**) Endogenous A1CF ubiquitination levels in HepG2 cells transfected with si-control or siTrim21. (**G** and **H**) Half-life measurements of endogenous A1CF in HepG2 cells (**G**), or overexpressed A1CF in Hepa 1–6 cells (**H**), cotransfected with TRIM21 WT or its LD form and treated with cycloheximide (CHX, 100 μg/mL) for the indicated time. (**I** and **J**) A1CF ubiquitination assays to identify the A1CF ubiquitination site in Hepa 1–6 cells cotransfected with either TRIM21 and A1CF (WT), single mutants (**I**), or double ubiquitination mutant (**J**), that were MG132 treated for 6 hours. (**K**) A1CF expression in Hepa 1–6 cells cotransfected with TRIM21 and A1CF-WT or indicated mutants. (**L**) Half-life measurements of A1CF in Hepa 1–6 cells cotransfected with TRIM21 and A1CF-WT or K302R mutant, treated with CHX for the indicated time intervals. ACTIN was used as a loading control.

To better understand the molecular basis of A1CF-mediated metabolic processes and to elucidate the mechanisms and upstream pathways that control its activity, we profiled A1CF’s interaction partners through an unbiased proteomics approach. Liver extracts from 4 adult mice were subjected to endogenous A1CF immunoprecipitation and analyzed by label-free LC-MS/MS shotgun proteomics. Interestingly, the E3 ubiquitin ligase TRIM21 was identified as the top interactor of A1CF and validated by co-IP (Figure 1, A and B; Supplemental Figure 1A; and Supplemental Table 1; supplemental material available online with this article; https://doi.org/10.1172/jci.insight.164694DS1). Another RNA binding protein (RBP) — RBM47, known to interact with A1CF (41) — was also identified in our proteomics analysis and validated by co-IP (Supplemental Figure 1B and Supplemental Table 1). Given that TRIM21 has been shown to exhibit K63- and K48-ubiquitin ligase activity, we hypothesized that this enzyme might control the ubiquitination of A1CF. To address this question, we first investigated whether A1CF is a substrate protein for polyubiquitination. Strong total and K63-linked ubiquitination signals were detected in mouse liver and in HepG2 cells when A1CF was immunoprecipitated (Figure 1, C and D). To determine whether A1CF polyubiquitination is mediated directly by TRIM21, the TRIM21 WT (TRIM21 WT) and its ligase-dead (LD) mutant form (TRIM21-LD: C16A, C31A, and H33W) (42) were coexpressed with A1CF in Hepa 1–6 cells. TRIM21 WT expression elevated A1CF polyubiquitination, while the coexpression with LD mutant retained the ubiquitination signal of untransfected cells (Figure 1E). Importantly, the TRIM21-mediated A1CF polyubiquitination was inversely correlated with reduced A1CF levels in 2 different cell lines (Figure 1E and Supplemental Figure 1C). Consistently, knockdown of TRIM21 in HepG2 cells, a cell line that coexpresses TRIM21 and A1CF (Supplemental Figure 1D), eliminated the endogenous A1CF polyubiquitination and increased its expression (Figure 1F). In concordance, the half-life of either endogenous or the overexpressed A1CF was reduced upon coexpression with TRIM21 WT in HepG2 or in Hepa 1–6 cells, while its enzymatic-inactive TRIM21-LD form had no impact (Figure 1, G and H). Next, we determined the site of TRIM21-mediated ubiquitination by searching the Phosphosite plus database, a resource depicting all curated posttranslational modifications for mouse A1CF. We found a total of 9 lysine residues as substrates for ubiquitination (Supplemental Figure 1E) (Phosphosite plus, http://www.phosphosite.org). Lysine (K) to arginine (R) point mutations identified K302 as the critical residue for A1CF ubiquitination by TRIM21 (Figure 1I). Reduction of A1CF-K302R ubiquitination was accompanied by increased A1CF expression (Figure 1I). Additional mutagenesis of the neighboring K249 — which also displayed reduced ubiquitination, although less pronounced — had no impact on A1CF protein levels (Figure 1, I–K). In concordance, K302R was resistant to ubiquitination-mediated degradation upon translation inhibition by cycloheximide treatment (Figure 1L). Collectively, these data reveal that the E3 ligase activity of TRIM21 is required for A1CF polyubiquitination and that K302 is the critical residue.

### TRIM21 expression is induced in steatohepatitis.

**Figure 2:**
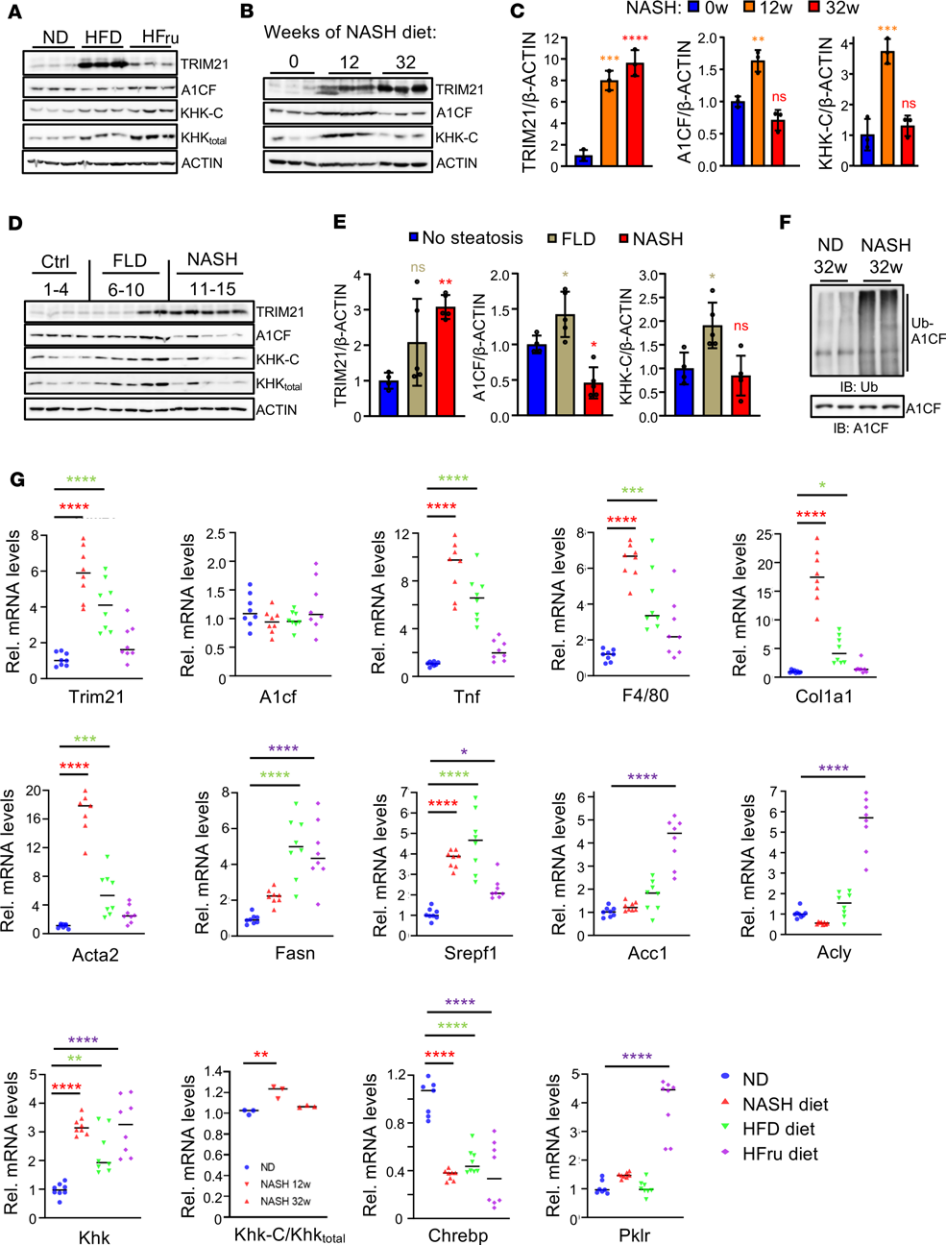
TRIM21 expression is induced in steatohepatitis. (**A** and **B**) Western blots showing the expression of indicated proteins in livers of mice fed a normal diet (ND), high-fat (HFD, 16 weeks), or high-fructose diet (HFru, 8 weeks) (**A**) or a NASH diet for the indicated periods (**B**), *n* = 3 replicates/group, 1 mouse/replicate). (**C**) Western blot signal quantification of **B** by densitometry. (**D**) Western blot of indicated proteins from liver biopsies of individuals without steatosis (Ctrl, *n* = 4), simple steatosis (FLD, *n* = 5), or NASH (*n* = 5). (**E**) Western blot signal quantification of **D** by densitometry. (**F**) Endogenous A1CF ubiquitination levels from livers of mice fed a chow (ND) or NASH diet for 32 mice weeks (*n* = 2 replicates per group, 1 mouse/replicate). (**G**) Relative mRNA levels of indicated genes in livers of mice fed with normal (ND), NASH diet (32 weeks), HFD (16 weeks), or HFru diet (8 weeks), *n* = 8 mice/group. The KHK splicing analysis is calculated as the ratio of KHK-C isoform expression in relation to total KHK in livers of mice fed ND or NASH diets at the indicated time points (*n* = 3 replicates/group, 1 mouse/replicate). In all statistical plots, data are expressed as mean ± SD; ****P < 0.0001; ***P < 0.001; **P < 0.01; *P < 0.05. *n* represents number of replicates, 1 mouse/replicate. For **C**, **E**, and **G**, the statistical analysis was carried out by 1-way ANOVA with Sidak’s post hoc analysis.

Transcript and protein expression data indicate that TRIM21 levels are low in healthy livers of mice fed a normal chow diet (ND). We investigated whether metabolic stress conditions and liver disease states affect the expression of TRIM21. In fasted and refed mice, hepatic TRIM21 levels were similar, and insulin or fructose treatments of hepatic cells had no impact on TRIM21 expression (Supplemental Figure 2, A–C). However, TRIM21 levels were profoundly increased in livers of obese mice on a high-fat diet (HFD, 60% fat) or a NASH diet (40% fat, 30% fructose diet, and 2% cholesterol) as compared with ND controls (Supplemental Figure 2A and Figure 2, A–C). Feeding mice a high-fructose diet (60% fructose for 8 weeks) led to a modest (approximately 1.8-fold) increase in TRIM21 expression, which was amplified in mice fed a high-fat and high-cholesterol diet containing fructose (NASH diet) (Figure 2, A–C). Importantly, in liver biopsies of patients without fatty liver disease (FLD) and with NASH (Supplemental Table 2), TRIM21 expression was markedly induced in steatohepatitis (Figure 2, D and E). TRIM21 induction was accompanied by a reduction of A1CF protein expression, in both mice and humans with NASH, and in HFD-fed mice (Figure 2, A–E). Consistent with the profound upregulation of TRIM21 levels, we observed increased A1CF ubiquitination in liver lysates of mice fed a NASH diet for 32 weeks compared with mice fed a ND (Figure 2F). Notably, steady-state A1CF mRNA levels were not reduced in any of the above disease conditions (Figure 2G and Supplemental Figure 2K), indicating that the observed A1CF reduction is not transcriptionally regulated but likely mediated by degradation through TRIM21. We also measured a reduction in KHK-C levels, consistent with our recent findings that A1CF regulates KHK-C expression through alternative splicing and showing that the decreased A1CF levels in both mice and humans with NASH are functionally relevant (Figure 2, A–E). Expression profiling in livers of mice with developing steatohepatitis revealed that, at the time when livers start to accumulate lipids and develop steatosis (12 weeks of NASH diet), A1CF and KHK are induced, while TRIM21 expression starts to increase (Figure 2, B and C). Importantly, at age 32 weeks, when the NASH pathophysiology, including inflammation and fibrosis, becomes more apparent (Supplemental Figure 2, D–J), we found that TRIM21 induction reaches a plateau and is negatively correlated with A1CF protein expression and, subsequently, of KHK-C, due to aberrant splicing (Figure 2, B–E). Furthermore, Trim21 mRNA induction was positively correlated with Khk levels, proinflammatory and fibrotic markers, an observation that was less pronounced for lipogenesis genes (Figure 2G). Similar results were obtained from liver biopsies of patients with FLD and NASH, with increased TRIM21 levels in NASH and reduced A1CF and KHK-C protein levels (Figure 2D). As expected, inflammatory and fibrosis markers were markedly increased in NASH livers, with only modest or no rises in DNL genes compared with FLD and healthy control livers (Supplemental Figure 2K). These data led us to hypothesize that, upon progression of fatty liver to NASH, TRIM21 is induced to degrade A1CF and thereby reduce KHK-C expression as a protective mechanism to suppress fructose-induced steatosis.

### TNF-α, fatty acids, ER stress, and oxidative stress induce TRIM21 in hepatocytes.

**Figure 3:**
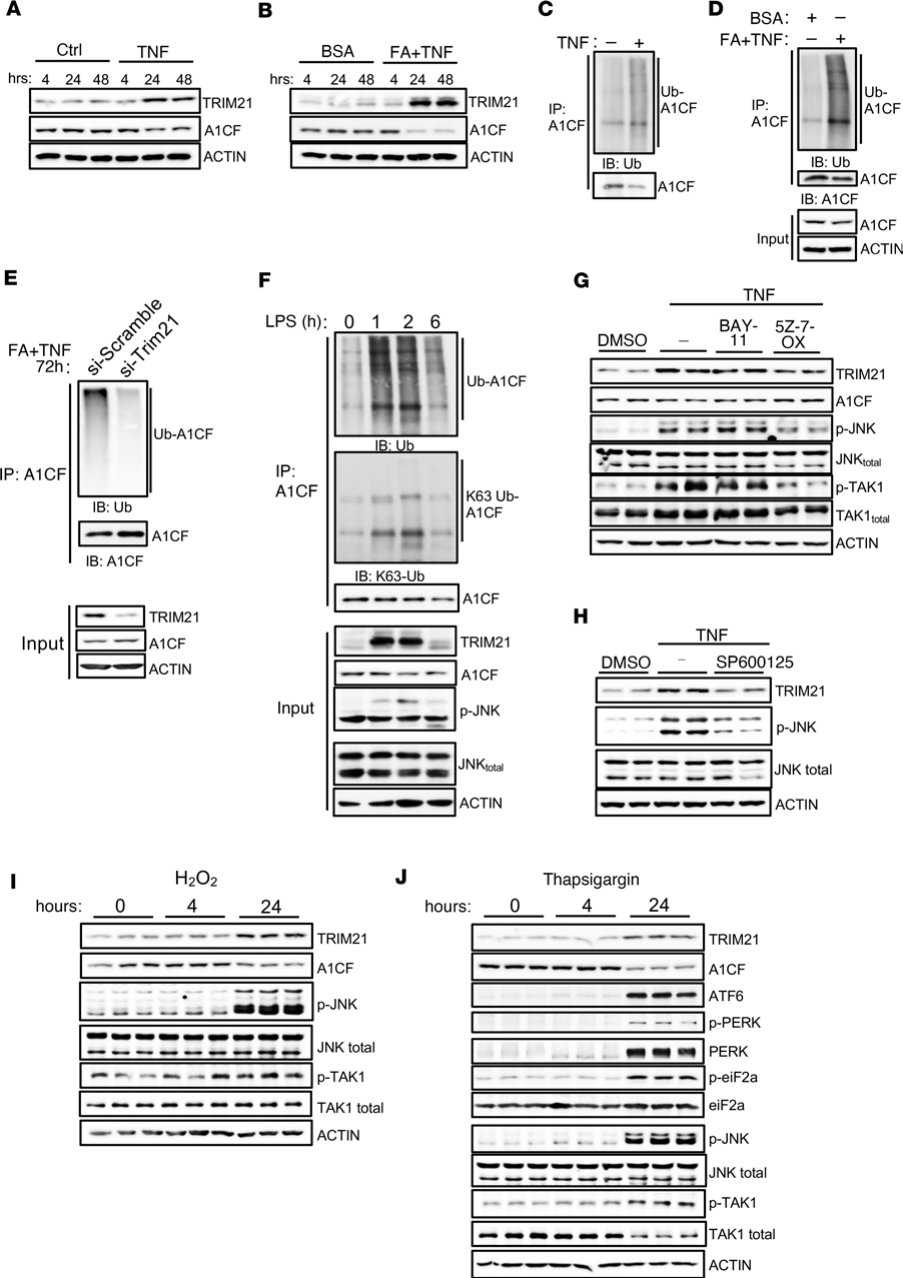
TNF and fatty acids, ER- and oxidative stress induce TRIM21 in hepatocytes. (**A** and **B**) Immunoblot analysis of TRIM21 and A1CF expression in HepG2 cells stimulated with either TNF-α (20 ng/mL) (**A**) or with TNF-α and fatty acids (**B**) (0.5 mM mixture of oleic and palmitic acid, FA+TNF) for the indicated time. (**C** and **D**) Endogenous A1CF ubiquitination levels in HepG2 cells stimulated for 48 hours with either TNF-α (**C**) or with TNF-α and FA (**D**). (**E**) Endogenous A1CF ubiquitination levels in HepG2 cells challenged with TNF-α and FA for 72 hours and transfected with si-Trim21 or Scramble (control) for 48 hours. Input levels show TRIM21-knockdown efficiency and A1CF levels. (**F**) Ubiquitination assay determining total and K63-linked endogenous A1CF ubiquitination in liver extracts from mice challenged with LPS (5 mg/kg) for the indicated time points (*n* = 2 livers pooled per time point). (**G**) Expression levels of the indicated proteins in HepG2 cells stimulated with TNF-α (20 ng/mL) for 48 hours in the presence of inhibitors for NF-κB (10 μM, BAY-11) or TAK1 (1 μM, 5z-7-oxozeaenol) (*n* = 2 replicates per time point). (**H**) Expression levels of indicated proteins in HepG2 cells that were pretreated with the JNK inhibitor SP600125 (5 μM) and stimulated with TNF-α (20 ng/mL) for 24 hours; vehicle (DMSO) was used as control. (**I** and **J**) Expression levels of indicated proteins in HepG2 cells stimulated with 1 mM hydrogen peroxide (H_2_O_2_) (**I**) or with 0.2 μM thapsigargin (**J**) for the indicated time points (*n* = 3 replicates per time point each).

To explore the molecular mechanisms underlying TRIM21 induction and A1CF protein degradation in FLD, we tested hepatic cells with factors that have been linked to NASH progression, such as high fructose, insulin, TNF-α, and fatty acids. As described above, stimulation of HepG2 cells with fructose and insulin had no effect (Supplemental Figure 2, A–C), whereas TNF-α stimulation induced TRIM21 and reduced A1CF protein expression (Figure 3A). Fatty acids (FA) efficiently induced lipid accumulation in hepatic cells (Supplemental Figure 3) and, in combination with TNF-α, potentiated the expression of TRIM21 (Figure 3B), while almost eliminating A1CF protein levels as compared with stimulation with TNF-α only (Figure 3, A and B). Importantly, TNF-α and FA stimulation induced A1CF polyubiquitination (Figure 3, C and D), while the knockdown of TRIM21 reduced it (Figure 3E). To investigate the ability of TNF-α to increase TRIM21 expression in vivo, we administered mice with LPS, which induces TNF-α production in Kupffer cells (KCs), the resident macrophages in the liver (43, 44). I.p. LPS administration activated hepatic JNK expression, providing evidence for efficient TNF-α stimulation of hepatocytes, and induced TRIM21 expression and A1CF polyubiquitination, but not the K63-linked, resulting in reduced A1CF protein levels (Figure 3F). Pretreatment of HepG2 cells with 5z-7-oxozeaenol, a TAK1 inhibitor, revealed that the TNF-α–mediated TRIM21 induction and A1CF protein degradation is TAK1 dependent (Figure 3G). Furthermore, pretreatment of HepG2 cells with the JNK inhibitor SP600125 also prevented the upregulation of TRIM21 upon TNF-α stimulation (Figure 3H), while inhibition of NF-κB activation via BAY-11 inhibitor had no effect (Figure 3G). These results suggest that induction of TRIM21 expression is mediated by TAK1 and JNK signaling under the stimulation of TNF-α. We further observed that exposing HepG2 cells to H_2_O_2_ or thapsigargin — known oxidative and ER-stressors, respectively — also induced TRIM21 and reduced A1CF expression (Figure 3, I and J). These findings show that the principal factors that have been associated with steatohepatitis progression in mice and humans — high levels of FA in combination with proinflammatory TNF-α as well as oxidative and ER stress — are potent and synergistic inducers of TRIM21 expression.

### Hepatic TRIM21 protects from fructose-induced steatosis via degradation of A1CF.

**Figure 4:**
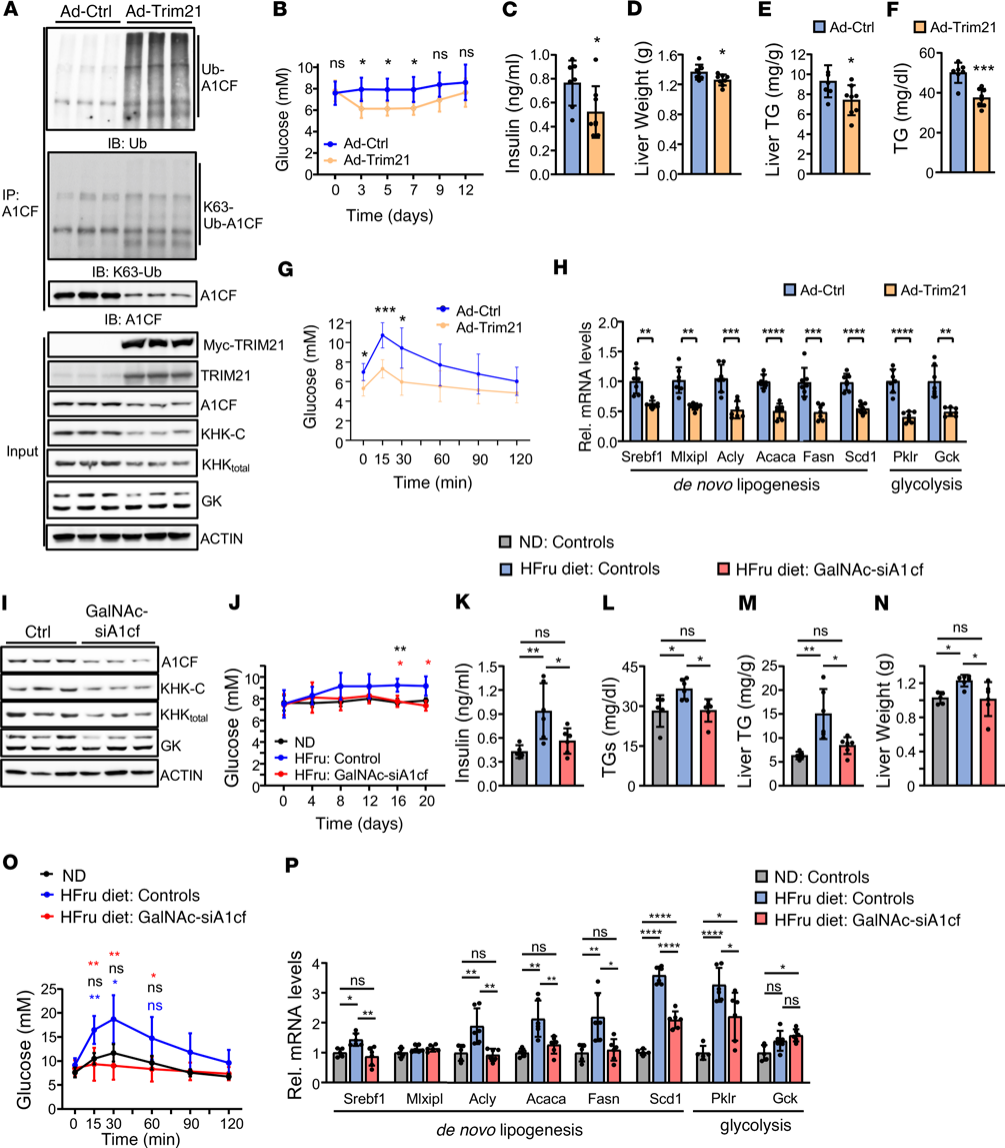
Hepatic TRIM21 protects from fructose-induced steatosis via degradation of A1CF. (**A**) Levels of total and K63-linked endogenous A1CF ubiquitination and input protein expression in liver extracts of Ad-Trim21 or Ad-Ctrl C57BL/6N mice fed a HFru diet for 4 weeks (*n* = 3 mice per group). (**B–H**) Random plasma blood glucose (**B**), plasma insulin levels (**C**), liver weight (**D**), liver triglyceride (TG) (**E**), plasma TG (**F**), fructose-tolerance test (3 g/kg fructose) (**G**), and relative hepatic mRNA expression of indicated genes (*n* = 7 mice per group) (**H**) from mice indicated as in **A**. (**I**) Western blot analysis of indicated proteins in livers of GalNAc-siA1cf and control injected 12-week-old C57BL/6N mice fed HFru diet for 4 weeks (*n* = 3 mice/group). (**J**) Blood glucose from C57BL/6N control mice fed a normal diet (ND) or GalNAc-siA1cf–injected and control mice fed a HFru diet for 4 weeks. (**K–P**) Plasma insulin (**K**), plasma TG (**L**), hepatic TG levels (**M**), liver weight (**N**), fructose tolerance test (3 g/kg Fru) (**O**), (blue asterisks. ND versus HFru diet control; black, ND versus HFru GalNAc-siA1cf; red asterisks, HFru control versus HFru GalNAc-siA1cf) and relative hepatic mRNA expression of indicated metabolic genes (**P**) from mice indicated as in **J**. Mice per group for **B**–**G**: Ad-Ctrl (*n* = 7), Ad-Trim21 (*n* = 8); for **J**–**P**: ND (*n* = 5), controls or GalNAc-siA1cf in HFru diet group (*n* = 6 each). *n* represents number of replicates, 1 mouse/replicate. In all statistical plots, data are expressed as mean ± SD; ****P < 0.0001; ***P < 0.001; **P < 0.01; *P < 0.05. Statistical analysis for **B**, **G**, **J**, and **O** was carried out by 2-way ANOVA with Sidak’s post hoc analysis; for **C**–**F** and **H** by *t* test; and for **K–N** and **P** by 1-way ANOVA with Sidak’s post hoc analysis.

To study the consequences and potential beneficial effects of TRIM21-mediated A1CF degradation and KHK-C suppression in livers, we overexpressed TRIM21 in the livers of mice fed a ND with 30% fructose supplementation in the drinking water for 4 weeks (high-fructose [HFru] diet). Recombinant adenoviral TRIM21 (Ad-Trim21) infection of mice increased hepatic TRIM21 expression to levels similar as those observed in mice on HFD or NASH diets (Figure 2, A–C; Figure 4A; and Supplemental Figure 4A). Hepatic TRIM21 overexpression strongly induced A1CF polyubiquitination, but not the addition of *K63*-linked ubiquitin chains (Figure 4A). These data demonstrate that TRIM21 promotes the K48-polyubiquitination and degradation of A1CF, thereby suppressing KHK-C and GK exon5+ isoform expression (Figure 4A and Supplemental Figure 4B). Phenotypically, after 12 days of hepatic TRIM21 overexpression, glycemia, plasma insulin levels, and liver weight were markedly lower compared with Ad-Ctrl–injected animals (Figure 4, B–D), most likely due to reduced fructose-mediated lipid accumulation in liver (Figure 4E), while whole-body and epididymal fat weight remained unchanged (Supplemental Figure 4, C–E). Plasma triacylglycerol (TG) levels were reduced (Figure 4F), while cholesterol and nonesterified fatty acids (NEFA) remained unchanged (Supplemental Figure 4, F and G). Furthermore, tolerance tests for fructose (FTT) and glucose (GTT) were improved in Ad-Trim21–injected mice compared with controls (Figure 4G and Supplemental Figure 4H). Gene expression profiling of Ad-Trim21 livers versus Ad-Ctrl controls demonstrated that the TRIM21-mediated A1CF degradation and inhibition of KHK-C expression reduced the expression of DNL, glycolysis, and gluconeogenesis genes, a finding that likely explains the steatotic improvement and enhanced glucose tolerance in mice overexpressing TRIM21 in the liver (Figure 4H).

To provide direct evidence that these metabolic improvements are mediated through A1CF degradation, A1CF was silenced in vivo using a chemically modified siRNA that was covalently conjugated to a trivalent N-acetylgalactosamine (GalNAc-siA1cf) for hepatocyte-specific uptake (Supplemental Figure 4I). S.c. administration of GalNAc-siA1cf twice weekly, for 3 weeks, resulted in an approximately 50% reduction of hepatic A1CF (Figure 4I and Supplemental Figure 4J). This modest decrease induced aberrant alternative splicing of *Khk* and *Gk* pre-mRNAs resulting in diminished *Khk-C* and *Gk* exon5+ isoform levels (Figure 4I and Supplemental Figure 4K). Hepatic *A1cf* silencing in mice fed a HFru diet for 4 weeks had no effect on body weight as compared with its controls, as well as to control mice fed a ND (Supplemental Figure 4L). However, glycemia, plasma insulin, triglycerides, cholesterol and NEFA, liver TG levels, epididymal fat, and liver weight were improved, at levels similar as ND-fed controls (Figure 4, J–N, and Supplemental Figure 4, L–Q). Furthermore, fructose and glucose stress tests, as measured by FTT and GTT, were also improved (Figure 4O and Supplemental Figure 4R). Of note, the expression of DNL genes in GalNAc-siA1cf–treated livers was 50% reduced and comparable with Ad-Trim21 livers of mice fed the same HFru diet for 4 weeks (Figure 4P). Collectively, these findings indicate that the steatotic improvements observed in Trim21-overexpressing livers were mediated by A1CF degradation, and strongly they support the conclusion that that high levels of TRIM21 in the liver exhibited similar metabolic improvements than in KHK-C deficiency mediated by A1CF silencing.

### TRIM21 improves steatosis in NASH through inhibition of fructose and lipid metabolism.

**Figure 5:**
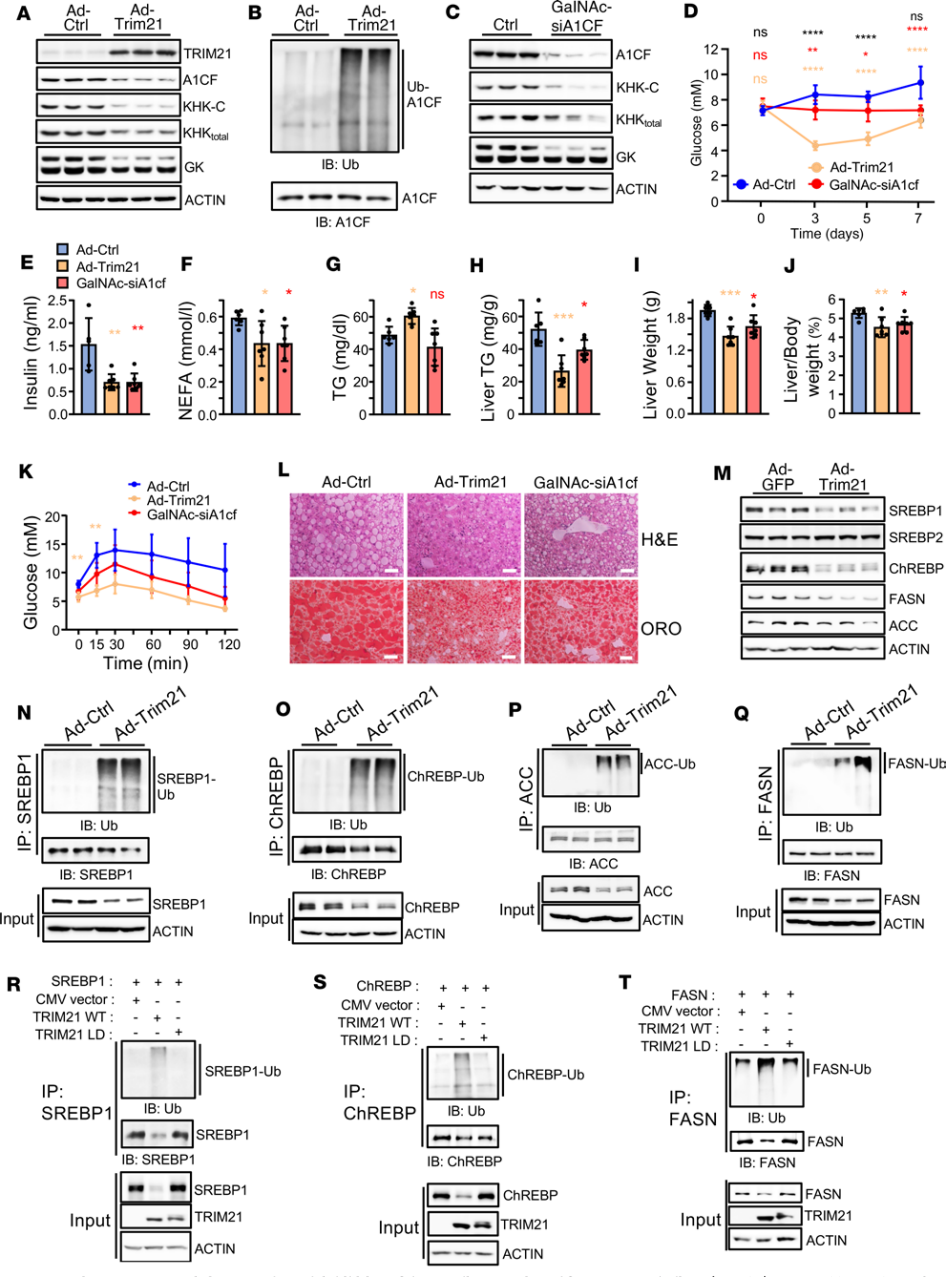
TRIM21 improves steatosis in NASH through inhibition of de novo lipogenesis and fructose metabolism. (**A** and **B**) Western blot analysis of indicated proteins (**A**) and total A1CF ubiquitination (**B**) from livers of mice fed a NASH diet for 20 weeks and injected with Ad-Trim21 or control adenovirus. (**C**) Western blot analysis of indicated proteins from livers of mice fed a NASH diet for 20 weeks and injected with GalNAc-siA1cf or control (*n* = 3). (**D**) Plasma blood glucose from C57BL/6N mice fed a NASH diet for 20 weeks and injected with Ad-Ctrl, Ad-Trim21, or GalNAc-siA1cf. Black asterisks, Ad-Trim21 versus GalNAc-siA1cf; red, Ctrl versus GalNAc-siA1cf; orange, Ctrl versus Ad-trim21. (**E–L**) Plasma insulin (**E**), NEFA (**F**), TG (**G**), liver TG (**H**), liver weight (**I**), ratios of liver/body weight (**J**), fructose-tolerance test (**K**), and H&E and Oil Red O (ORO) staining of liver sections (**L**) from mice indicated as in **D**. (**M**) Western blot analysis of indicated lipogenesis genes in livers of mice indicated as in **A**. (**N–Q**) Endogenous ubiquitination of SREBP1 (**N**), ChREBP (**O**), ACC (**P**), and FASN (**Q**) in livers of mice indicated as in **A** (*n* = 2). (**R**–**T**) Ubiquitination assays in Hepa 1–6 cells cotransfected with TRIM21 WT or its ligase-dead (LD) form and SREBP1 (**R**), ChREBP (**S**), and FASN (**T**). Input shows the expression levels of the indicated proteins. Scale bars: 50 μm. Mice/group for **D**–**K**: *n* = 6 for controls and *n* = 7 for Ad-Trim21 or GalNAc-siA1cf. *n* represents number of replicates, 1 mouse/replicate. In all statistical plots, data are expressed as mean ± SD; ****P < 0.0001; ***P < 0.001; **P < 0.01; *P < 0.05. Statistical analysis for **D** and **K** was carried out by 2-way and for **E**–**J** by 1-way ANOVA with Sidak’s post hoc analysis.

Based on the observed metabolic improvements of Ad-Trim21–injected mice, and due to the association between fructose consumption and NASH progression, we decided to further elucidate the function of hepatic TRIM21 in the pathogenesis of NASH. We overexpressed TRIM21 in mice fed a NASH diet for 20 weeks, when TRIM21 levels have not reached the plateau observed at later stages (32 weeks) in well-developed NASH (Figure 2, B and C). TRIM21 overexpression increased A1CF ubiquitination and reduced A1CF, KHK-C, and GK isoform expression (Figure 5, A and B, and Supplemental Figure 5A). Given that A1CF-mediated KHK-C expression promotes fructose metabolism and, ultimately, hepatic steatosis at early stages of NASH development, we also investigated A1CF’s role as a contributor to NASH progression. Knockdown of A1CF using GalNAc-siA1cf in 20-week-old mice that were fed a NASH diet induced a similar reduction in KHK-C and GK isoforms than overexpression of TRIM21, due to splicing defects caused by A1CF depletion (Figure 5C and Supplemental Figure 5A). Total body and epidydimal fat weight were reduced only in Ad-Trim21 mice, while glycemia was improved upon both TRIM21 overexpression and A1CF silencing (Supplemental Figure 5, B–D, and Figure 5D). Similarly, plasma insulin and NEFA levels were also improved (Figure 5, E and F). Plasma TGs were induced only in Ad-Trim21 mice (Figure 5G), while liver TG and ratios of liver/body weight were reduced and while fructose tolerance and insulin sensitivity were improved in mice with hepatic TRIM21 overexpression and A1CF knockdown compared with controls (Figure 5, H–K, and Supplemental Figure 5E). Histological analysis of Oil Red O–stained liver sections from these mice confirmed the improvement in steatosis (Figure 5L).

Despite the several common phenotypic and biochemical outputs of hepatic TRIM21 overexpression and A1CF silencing, we noticed that the improvement in steatosis was more profound in Ad-Trim21 mice compared with GalNAc-siA1cf–treated mice. This finding indicated that TRIM21 might have additional and A1CF-independent metabolic targets that influence hepatic lipogenesis. To search for candidate proteins that could be targeted for degradation by Trim21, we analyzed the expression of numerous regulators of DNL in livers with TRIM21 overexpression. Interestingly, we found that protein levels of SREBP1 and ChREBP, 2 transcription factors that play critical roles in hepatic FA synthesis and steatosis, as well as FASN and ACC, 2 rate-limiting metabolic enzymes of DNL, were reduced in TRIM21-overexpressing livers compared with controls (Figure 5M). In human NASH liver biopsies with increased TRIM21 expression, we also measured decreased SREBP1, FASN, and ACC proteins levels, which were accompanied by unchanged steady-state mRNA levels (Supplemental Figure 2K and Supplemental Figure 5F). Furthermore, this regulation was also observed in HepG2 cells that were treated with FA and TNF-α (Supplemental Figure 5G). Of note, in human biopsies of patients with NASH, we measured reduced ChREBP mRNA levels as compared with patients with NAFLD, and the lower ChREBP mRNA levels in NASH may contribute to the reduction of ChREBP protein levels. In concordance, in vivo overexpression of TRIM21 in livers increased the ubiquitination of endogenous SREBP1 and ChREBP, and of ACC and FASN enzymes (Figure 5, N–Q). To determine whether the ubiquitination is mediated directly by TRIM21, the TRIM21 WT and its LD form were coexpressed with SREBP1, ChREBP, and FASN in Hepa 1–6 cells. TRIM21 expression elevated their ubiquitination and almost eliminated their expression, whereas coexpression of the TRIM21-LD mutant had no effect (Figure 5, R–T). In contrast, we found no evidence that TRIM21 ubiquitinates other important metabolic regulators in liver such as SREBP2, AKT1/2, KHK, JNK, and PEPCK (Supplemental Figures 5, H–L).

TRIM21 has been shown to function as a negative regulator of oxidative stress by ubiquitylating p62 at lysine(K)7 via K63-linkage, abrogation of p62 oligomerization, sequestration of KEAP1, and activation of NRF2 (39, 42). Although in Trim21-overexpressing livers we have found a moderate increase in K63-linked p62 ubiquitination and an increase in ROS accumulation, results that are consistent with previous studies, our analysis shows that the p62/KEAP1/NRF2 axis was not dysregulated, since the antioxidant gene expression was mostly induced due to potential compensation (Supplemental Figure 5, M–O). Furthermore, we measured increased plasma ALT, inflammatory markers, and JNK/TAK1 signaling upon Trim21 overexpression, and this was not observed in A1CF-silenced livers (Supplemental Figure 5, P–S). Collectively, these studies support a model whereby high levels of TRIM21 in the liver act as a counterregulatory force of steatosis development by regulating critical transcriptional and posttranscriptional factors as well as enzymes of DNL but also induce an inflammatory response that is independent of A1CF degradation.

### Trim21 silencing exacerbates steatosis in NASH.

**Figure 6:**
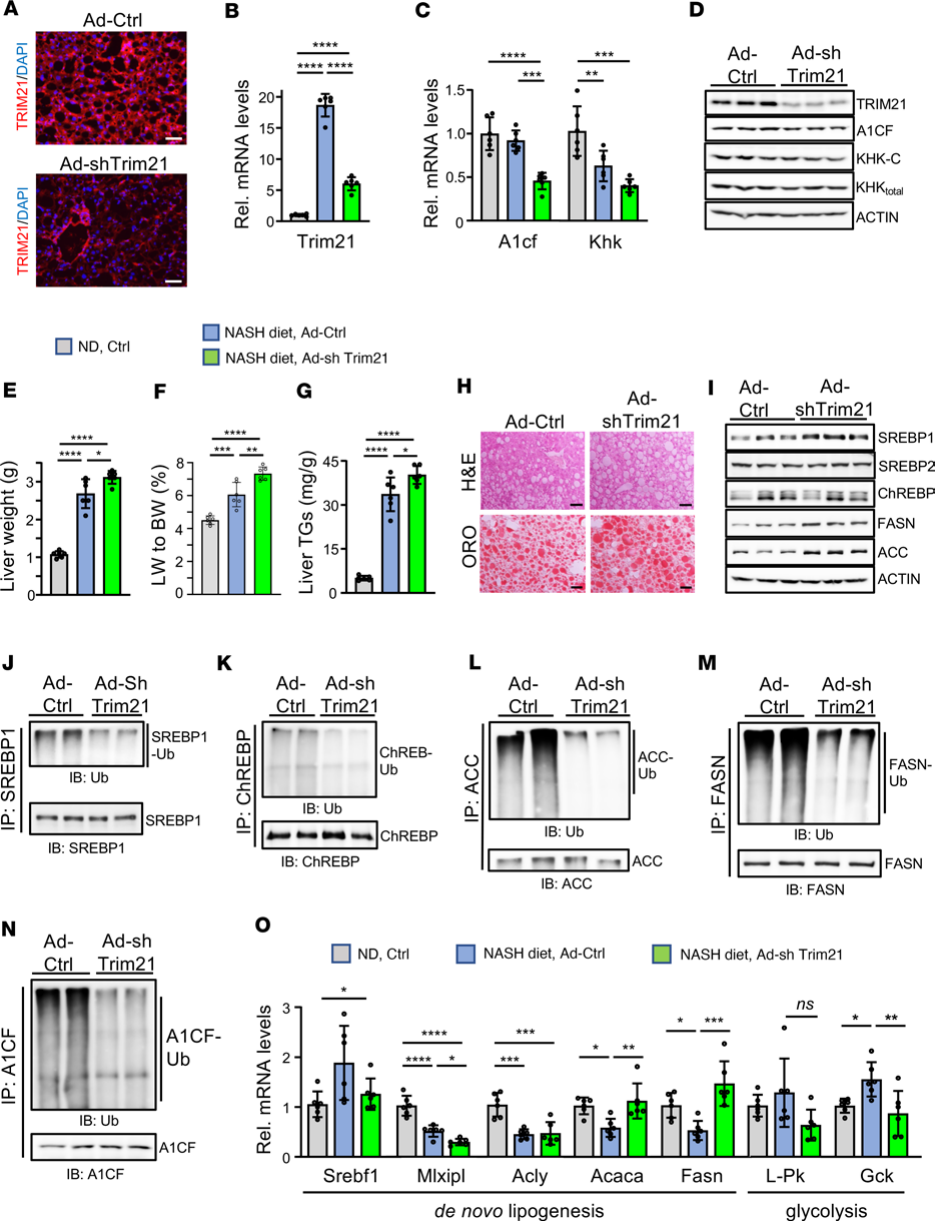
TRIM21 silencing exacerbates steatosis in NASH. (**A**) Immunostaining using anti-TRIM21 in liver sections from Ad-shTrim21 or Ad-Ctrl C57BL/6N mice fed a NASH diet for 30 weeks. (**B** and **C**) Relative hepatic mRNA expression of Trim21 (**B**), and A1cf and Khk (**C**). (**D**) Western blot analysis of indicated proteins (*n* = 3 mice per group). (**E**–**I**) Liver weight (**E**), ratios of liver/body weight (**F**), liver TG (**G**), H&E and Oil Red O staining of liver sections (**H**), and immunoblot analysis of indicated lipogenesis genes (**I**) (*n* = 3 mice per group) from mice indicated as in **A**. (**J–N**) Endogenous ubiquitination of SREBP1 (**J**), ChREBP (**K**), ACC (**L**), FASN (**M**), and A1CF (**N**) from liver extracts from mice indicated as in **A** (*n* = 2). (**O**) Relative mRNA expression of de novo lipogenesis and glycolysis genes from mice indicated as in **A**. Scale bars: 50 μm. *n* represents number of replicates, 1 mouse/replicate. In all statistical plots, data are expressed as the mean ± SD; ****P < 0.0001; ***P < 0.001; **P < 0.01; *P < 0.05. Statistical analysis for **B**, **C**, **E–G**, and **O** were carried out by 1-way ANOVA with Sidak’s post hoc analysis, *n* = 6 mice per group.

To further confirm the function of TRIM21 in hepatocytes and in NASH pathogenesis, we knocked down TRIM21 by approximately 60% in livers of mice fed a NASH diet for 30 weeks using a recombinant adenovirus-mediated delivery of shTrim21 for 9 days (Figure 6, A and B). Total body fat and epidymidal fat weights as well as plasma glucose levels remained unchanged in NASH mice with Trim21-silenced livers (Supplemental Figure 6, A–D). Unexpectedly, we measured reduced mRNA levels of *A1cf* and *Khk* but similar A1CF and KHK-C protein levels in livers of *Trim21*-knockdown mice compared with controls (Figure 6, C and D). This finding was in concordance with the similar ability of Trim21-silenced and control livers to convert fructose to glucose, as measured by a FTT (Supplemental Figure 6E) and consistent with the sustained KHK protein levels (Figure 6D). In contrast to Trim21 overexpression, silencing of *Trim21* resulted in increased liver weight and steatosis, as evaluated by liver triacylglyceride measurements and Oil Red O staining (Figure 6, E–G). Furthermore, we measured increased protein levels of SREBP1, FASN, and ACC upon Trim21 silencing, consistent with the observed enhanced steatosis (Figure 6H). Unexpectedly, the protein levels of ChREBP remained unchanged, possibly due to reduced mRNA expression in TRIM21-silenced livers by an unknown mechanism (Figure 6, I and O). Although the Trim21-overexpression data suggest a TRIM21-mediated ubiquitination-dependent degradation control of ChREBP, it is likely that the transient and incomplete Trim21 silencing we used in this study is inefficient to regulate ChREBP expression and contribute to the exaggerated steatosis. In concordance, we have found a mild reduction of its bona fide glycolysis gene targets (Figure 6O).

To test if changes in lipogenic protein levels could be explained by a reduced degradation rate in TRIM21-silenced livers, we quantified their ubiquitination levels. Indeed, we detected lower endogenous ubiquitination signals of SREBP1, ChREBP, ACC, FASN, and A1CF in livers of mice injected with Ad-shTrim21 compared with Ad-Ctrl–treated mice (Figure 6, J–N). Interestingly, the transcript levels of Acc and Fasn were elevated upon Trim21 silencing (Figure 6O), most likely because of increased SREBP1 protein levels.

Lastly, we explored the effect of Trim21 knockdown on the inflammation in response to a NASH diet. Transient hepatic Trim21 silencing resulted in a moderate rise in inflammatory (TNF-α, F4/80) and fibrosis (Col1a1) markers, consistent with elevated steatosis, though it resulted in no changes in αSMA expression, JNK/TAK1 signaling, or plasma ALT levels in livers of NASH mice in which Trim21 was silenced compared with controls (Supplemental Figure 6, F–K).

Together, the results from *Trim21*-knockdown experiments further strengthen the notion that TRIM21 activation during chronic inflammatory and metabolic stress dampens metabolic pathways, leading to the development of hepatic steatosis.

## Discussion

In this study, we have identified TRIM21 E3 ubiquitin ligase as a crucial suppressor of DNL- and fructose-mediated steatosis in the liver. We show that TRIM21 is induced in livers of humans and mice with NASH and that TNF-α and FA, the principal promoters of NASH pathogenesis, activate TRIM21 expression in hepatocytes through TAK1/JNK signaling. Upon activation, TRIM21 ubiquitinates and degrades key positive regulators of lipogenesis that act at different levels of gene regulation, thereby counteracting excessive accumulation of hepatic triglycerides during metabolic stress.

We show that toxic ROS, TNF-α, and ER stress and lipotoxicity, all hallmarks of NASH development, can act as potent inducers of TRIM21 expression in hepatocytes. TRIM21 (alternatively known as Ro52), is a E3 ubiquitin ligase that promotes K48- and K63-linked ubiquitination of various proteins by regulating their proteasomal degradation and cascade activation, respectively (45–51). TRIM21 was originally implicated in innate immunity, autoimmune responses, and cancer (52–54); however, recent studies have suggested a role in cancer metabolism through ubiquitination and degradation of enzymes and signaling regulators (55–59). These in vitro–based findings suggest a tumor-suppressor role of TRIM21 by inhibiting metabolic pathways that fuel nucleic acid synthesis, cell growth, and proliferation. Investigations of other members of the TRIM family have suggested that they contribute to the control of inflammation and metabolic homeostasis of chronic liver diseases (38–40, 60).

Our study shows that TRIM21 overexpression triggers ubiquitination and degradation of SREBP1 and ChREBP, 2 central transcription factors that constitute the major activating pathway of DNL. In contrast, upon transient Trim21 silencing, only the protein expression of SREBP1 was inversely correlated, while ChREBP expression was unchanged. Therefore, although our Trim21 overexpression data in vivo and in vitro indicate that ChREBP is a direct target for degradation, our transient sh-mediated knockdown data cannot conclude that ChREBP acts in synergism with SREBP1 to increase steatosis; we thus hypothesize that the exaggeration of steatosis is mainly due to the activation of SREBP1 and FASN. Future studies using long-term hepatocyte-specific Trim21-KO models are needed to resolve this difference. SREBP and ChREBP pathways are activated by increased insulin signaling and increased glucose concentrations, respectively — both induced by feeding (61, 62). SREBP1 is activated by downstream insulin signaling events involving the PI3K/ PKB pathway, and activation of LXR, which upon heterodimerization with the retinoid X receptor (RXR), activates transcription of SREBP1c (63). ChREBP activation appears to be stimulated by a number of metabolites generated during glycolysis and ultimately is mediated by dephosphorylation of PKA or AMPK phosphorylation (64). Both SREBP1 and ChREBP act synergistically to activate rate-limiting enzymes such as ACC1 and FASN that catalyze the synthesis of intermediates for FA synthesis. Whereas many studies have elucidated the mechanisms by which SREBP1 and ChREBP activate lipogenesis, much less is known about how this hyperactive metabolic pathway is downregulated to prevent pathologic hyperactivity.

FBW7 ubiquitin ligase interacts with nuclear SREBP1 and enhances its ubiquitination and degradation through phosphorylation of T426 and S430 by GSK-3. FBW7 has been shown to degrade SREBP family transcription factors (37). Inactivation of endogenous Fbw7 results in SREBP 1 and 2 protein stabilization and enhanced expression of their target genes, influencing synthesis of cholesterol and FA as well as enhanced receptor-mediated uptake of LDL (37). Interestingly, whereas FBW7 seems to induce degradation of SREBP1 and 2, we found that TRIM21 only ubiquitinates and degrades SREBP1. Similarly, the E3 ligase SMURF2 has been identified to promote ChREBP ubiquitination and degradation via the proteasome pathway and thereby influence aerobic glycolysis, oxygen consumption, and cell proliferation in cancer cells (36). Although those 2 ubiquitin ligases have been suggested as regulators of FA metabolism, their role in vivo and their contribution in the context of NAFLD and NASH development has not been studied. Nevertheless, considering that their expression is not induced in these disease conditions (65), we may predict that those ubiquitin ligases are likely not involved in the physiological turnover of their targets.

It is also interesting to note that TRIM21 directly degrades ACC1 and FASN, key lipogenic enzymes that are activated by SREBP1c. Both lipogenic enzymes are activated in patients with NAFLD (66), where ACC, the rate-limiting step in FA synthesis, catalyzes the carboxylation of acetyl-CoA to malonyl-CoA, and FASN activity, responsible for the final step of DNL, converting acetyl-CoA and malonyl-CoA to palmitate. Both enzymes are also critical for steatosis development in insulin-resistant states, and inhibitors of ACC and FASN are currently in clinical development for NASH (67–70).

Dietary fructose transcriptionally induces the expression of KHK-C (16, 31, 71), the rate-limiting enzyme of fructolysis, in mice and humans (72). Our previous findings have uncovered an additional level of KHK-C regulation through the RNA-binding protein A1CF as the main alternative splicing regulator of KHK-A/C splicing in the liver. A1CF antagonizes the action of HNRNPH1/2 RBP, responsible for the generation of KHK-A, thereby promoting the expression of the high-activity KHK-C isoform. Genetic ablation of *A1cf* markedly reduced the expression of the KHK-C isoform and protected mice from fructose-induced metabolic disease (30), thereby phenocopying KHK deficiency (16, 29, 31). In this study, we report an upstream mechanism of KHK-C regulation through TRIM21-mediated A1CF ubiquitination and degradation that inhibits KHK-C and establishes a protective mechanism suppressing fructose-induced steatosis.

We further demonstrated in vivo that hepatocyte-specific overexpression of TRIM21 attenuated steatosis in mice fed with a HFru or NASH diet, while conversely, TRIM21 silencing markedly exacerbated it. In vivo silencing of A1cf via administration of GalNAc-siRNAs reduced lipogenic gene expression and liver steatosis and phenocopied the effects of pharmacologic inhibition of KHK (73, 74), making it a candidate therapeutic target for ameliorating steatosis during NASH progression.

Interestingly, in addition to the beneficial effects on lipogenic gene expression through degradation of A1CF, SREBP1, FASN, and ACC, we found that overexpression of Trim21 also ubiquitinated p62, which has previously been linked to ROS accumulation as a result of Nrf2 suppression (42, 39). The increase in ROS is consistent with increased markers of inflammation and fibrosis that were independent of A1CF. Interestingly, our analysis showed induction of antioxidant gene expression in Ad-Trim21 livers. This induction suggests a compensatory mechanism for reducing ROS accumulation. Although the Trim21/p62/Keap/Nrf2 axis in Ad-Trim21 NASH livers seem not overtly dysregulated, it will be important to investigate in more depth this mechanism upon long-term Trim21 overexpression or silencing and to further investigate if other TRIM21 targets contribute to this phenotype. Strikingly, the inflammatory/fibrotic response was also induced by TRIM21 silencing, likely because of the elevated steatosis, which may suggest TRIM21-independent elevation of inflammation resulted by the adenovirus infection or via other unknown mechanisms. Another limitation of our study is the relatively short-term overexpression and incomplete silencing of TRIM21 in NASH livers; therefore, longer-term manipulations of TRIM21 expression before NASH onset and after the manifestation of a full-blown phenotype will be important to assess the immune consequences of TRIM21 induction during prolonged liver steatosis.

Our findings establish a pathway of immune-metabolic crosstalk between F4/80^+^ KCs and hepatocytes that drive the metabolic reprogramming of hepatocytes, which includes the dampening of harmful effects of fructose metabolism as well as lipogenic metabolism during the transition of steatosis to steatohepatitis. This pathway is subdued in unstressed healthy conditions but becomes activated at the time of KC expansion and TNF-α induction in steatotic liver disease models (44, 75, 76). Interestingly, experimental hepatic overexpression of TRIM21 during early stages of NASH development (~20 weeks in a NASH diet) was able to ameliorate liver steatosis, suggesting that endogenous TRIM21 levels are not saturated and that pharmacological activation of TRIM21 might be an approach to control hepatic lipid accumulation. Additional and future studies are warranted to identify the complete TRIM21 ubiquitination target network and to investigate the long-term and dose-dependent activation and silencing of TRIM21 in hepatocytes as well as KCs. Such studies will shed insight if TRIM21 or downstream targets have the potential to become candidates for NASH therapeutics.

## Methods

### Human liver samples.

Human liver biopsies were obtained during the work-up of liver disease diagnostics in the outpatient clinic of the Division of Gastroenterology and Hepatology, University Hospital Basel, Basel, Switzerland. Detailed information about individuals used can be found in Supplemental Table 2.

### Mice and diets.

Mice on a C57BL/6N background (Janvier Labs) were housed in a pathogen-free animal facility at the Institute of Molecular Health Sciences at ETH Zurich and maintained in grouped cages in a temperature-controlled room (22°C), with humidity at 55% on a 12-hour light/dark cycle (light on from 06:00 to 18:00 hours). The Ad-Cmv-Trim21, Ad-sh-Trim21, or Ad-Ctrl mice were generated by i.v. injection with 3 × 10^9^ plaque-forming units of AAV-Cmv-huTRIM21, AAV-U6-shTrim21, or AAV-null, respectively. For *A1cf* silencing in liver, a chemical-modified and N-acetylgalactosamine conjugated siRNA (GalNAc-siA1cf) (sequence: 5’-GUGAAAAUCCUGUACGUAA-3’), provided by Alnylam, was administered s.c. twice weekly for 3 weeks at the concentration of 3 mg/kg. In this study, mice were fed either a standard chow diet (ND: catalog 3437 from KLIBA NAFAG, 18.5% protein, 4.5% fat, 38% starch), HFru diet (catalog TD.89247 from Envigo-Tekland Diet, 18.3% protein, 5.2% fat, 60.4% fructose), or high fructose in drinking water (30% fructose in water, MilliporeSigma, F0127). NASH diet included D09100310 from Research Diets (22.5% protein, 40% fat, 20% fructose, and 2% cholesterol), HFD (U8978 Version 19 from SAFE Complete Care Competence: 19.84% protein, 35.92% fat, 18% sucrose), and water ad libitum. Unless otherwise indicated in the figures and figure legends, all experiments were performed in randomly chosen age-matched male mice. Typically, each experiment was performed in cages of 5 individual mice.

### Adenovirus production and injection into mice.

For TRIM21 overexpression, the human TRIM21 cDNA (NM_003141) tagged by Myc-DDK was provided by Origene (RC202088) and subcloned under the CMV promoter into the adenovirus expression vector pVQAd CMV K-NpA from Viraquest (Ad-Trim21). This vector harbors GFP that is driven from an independent locus. Control adenovirus was based on the same vector backbone including GFP (Ad-Ctrl). For hepatic *Trim21* silencing, a retroviral pRS plasmid containing a 29-mer shRNA–specific for mouse Trim21: GATCG tgtctgacaataaggagaggtttagtaat TCAAGAG attactaaacctctccttattgtcagaca TTTTTT GAA, was provided by Origene (TR514859) and was subcloned under the U6 promoter into the AAV expressing vector pVQAd AscI-NpA from Viraquest (Ad-shTrim21). The adenoviral infection of mice was conducted through a single tail vein injection of 3 × 10^9^ pfu in a final volume of 0.2 mL, diluted in saline.

### Cell culture, transfection, treatment, and pharmacological inhibition.

The HepG2 (ATCC, HB-8065), Hela (ATCC, CCL-2), Hepa1-6 (ATCC, CRL-1830), C2C12 (ATCC, CRL-1772), 3T3L1 (ATCC, CL-173), and HEK293 (ATCC, CRL1673) cell lines were propagated in high glucose DMEM with 10% FBS, supplemented with 100 units/mL penicillin and 100 mg/mL streptomycin from Thermo Fisher Scientific. Cells were maintained at 37°C in a humidified incubator with 5% CO_2_ in air. For transient transfection, overexpression vectors or siRNAs were transfected into cells using Lipofectamine2000 or Lipofectamine RNAiMAX, respectively, according to Invitrogen instructions. The protein overexpression or knockdown efficiency were determined by Western blotting. HepG2 cells were treated with a FA mixture of 0.5 mM oleic and palmitic acid conjugated in FA-free BSA, 100 nM insulin, 5 mM fructose, 20 ng/mL TNF-α, 1 mM H_2_O_2_, or 0.2 μM thapsigargin from MilliporeSigma for the times indicated in figure legends. For pharmacological inhibition of TAK1 or NF-κB activation, HepG2 cells were serum starved overnight; pretreated for 3 hours with 5z-7-oxozeaenol (1 μM), SP600125 (5 μΜ), or BAY-11-7082 (10 μM); and then stimulated with TNF-α (20 ng/mL) for the times indicated in figure legends.

### Plasmids and mutagenesis.

The plasmids included: pLenti-msA1CF-Myc–expressing mouse A1CF (NM_001081074) tagged with Myc (MR224176L1), pRS-ms-shTRIM21 (TR514859), and the PCMV6-huTRIM21-expressing human TRIM21 cDNA (NM_003141) tagged by Myc (RC202088), provided by Origene. PCMV6-huTRIM21 LD plasmid (TRIM21-LD mutant) was generated as described before (42). Briefly, the point mutations included: C16A, C31A, and H33W, which were introduced in PCMV6-huTRIM21 plasmid through site-directed mutagenesis, using the following primers: for C16A: 5′-GGAGGTCACAGCCCCTATCTGCCTGGACCCCTTCG-3′ and 5′-GCAGATAGGGGCTGTGACCTCCTCCCACATC-3′; for C31A/H33A: 5′CATCGAGGCTGGCGCCAGCTTCTGCCAGGAATGC-3′ and 5′GAAGCTGGCGCCAGCCTCGATGCTCACAGGCTC -3′.

For A1CF mutants, the K6R, K62R, K114R, K249R, K302R, K440R, K482R, K502R, and K511R point mutants (lysine-to-arginine substitution) were introduced through site-directed mutagenesis using the primers listed in Supplemental Table 3. The following plasmids were purchased from Addgene: His-tagged human FASN (107138), FLAG-tagged human SREBP1c (26802), and cMyc-tagged mouse ChREBP (39235) cloned in pcDNA3.1 vector.

### Antibodies.

The antibodies against β-actin (1:1,000, 4970), Myc (1:1,000, 2272), phosphorylated JNK/SAPK (Thr183/Tyr185) (1:1,000, 9251), JNK/SAPK (1:1,000, 9252), phosphorylated TAK1 (Thr184/187) (1:1,000, 4508), TAK1 (1:1,000, 4505), phosphorylated PERK (Thr980) (1:1,000, 3179), PERK (1:1,000, 3192), phosphorylated eIF2a (Ser51) (1:1,000, 3398), eIF2a (1:1,000, 5324), AKT (1:1,000, 9272), FA Synthase-FASN (1:1,0000, 3180), Acetyl-CoA Carboxylase-ACC (1:1,000, 3662), and p62/SQSTM1 (1:1,000, 5114) were purchased from Cell Signaling Technology. Antibodies against TRIM21/Ro/SSA (1:1,000, 25351), KHK (1:15,000, 377411), Ub (1:1,000, 8017), HNF4a (1:500, 8987), and SREBP1 (1:1,000, 13551) were purchased from Santa Cruz Biotechnology Inc. The following antibodies were purchased from Abcam: Glycerol kinase (GK) (1:1,000, 126599), RBM47 (1:1,000, 167164), ATF6 (1:1,000, 122897), αSMA (1:100, 5694), and PEPCK/PCK1 (1:10,000, 70358). Antibodies against K63-linkeage–specific polyubiquitin (1:500, 14-6077-82) and F4/80 (1:100, 14-4801-82) were purchased from eBioscience, KHK-C–specific antibody from SAB Biotech (1:1,000, 21709), A1CF (1:2,000, PA5-60608), and SREBP2 (1:2,000, PA5-88943) from Thermo Fisher Scientific; ChREBP (NB400-135) from Novus Biologicals; and FLAG-M2 (F1804) from Sigma-Aldrich. The secondaries antibodies against mouse IgG-HRP (1:5,000, 401253) and rabbit IgG-HRP (1:5,000, 401393) were purchased from Calbiochem.

### Chemicals.

The following chemicals were purchased from Sigma-Aldrich: cyclohexamide (C7698), LPS (L2880), BSA (05479), sodium oleate (O7501), sodium palmitate (P9767), BSA FA-free (A8806), insulin solution-human (I9278), fructose (F0127), glucose (49139), TNF-α (SRP3177), 5z-7-oxozeaenol (O9890), SP600125 (S5567), BAY-11-7082 (B5556), hydrogen peroxide (H_2_O_2_, 216763), Sirius red (Direct red 80, 365548), Oil Red O (O0625), Trizol (T9424), and N-ethylmaleimide (E3876). DAPI (D3571), Dynabeads Protein G (10003D), lipofectamine 2000 (11668-019), and RNAiMAX (13778-075) were from Invitrogen. The proteosome inhibitor MG132 (BML-PI102-0005) was from Enzo Life Sciences. The RNAse inhibitor (M0314) was from NEB. The DNaseI recombinant RNAse free (04716728001), complete EDTA-free proteases inhibitor cocktail tablets (05056489001), and PhoSTOP phosphatase inhibitors (04906837001) were from Roche. The Bradford Ultra reagent (BFU05L) was from Expedeon. The nonfat dry milk (A0830) was from AppliChem, and the ECL chemiluminescent kit (32209) was from Thermo Fisher Scientific.

### GTTs, FTTs, and insulin tolerance tests (ITTs).

I.p. tolerance tests were conducted in 6-hour fasted male mice. Glucose (2 g/kg), fructose (3 g/kg), and insulin (0.75U/Kg) from MilliporeSigma were dissolved in PBS and administered via i.p. injection. Blood glucose levels were measured using Contour Next glucose meter from tail snips prior to injection (baseline measurement) and after 15, 30, 60, 90, and 120 minutes.

### Blood collection and plasma measurements.

Blood was collected using nonheparinized capillaries by retro-orbital or tail vein bleeding into tubes containing 5 mM EDTA as an anticoagulant. The plasma was separated by centrifugation at 8,000*g* at 4°C for 8 minutes and immediately was frozen at –80°C. The plasma Triglycerides/Glycerol Blanked (catalog 11877771) and cholesterol (catalog 11489232) levels were measured using the Cfas standards (Calibrator for Automated Systems, 10759350190) from Roche, plasma insulin using Alpco (catalog 80-INSRTU-E10), NEFA using Wako diagnostics (catalogs 91696 and 91898), and ALT using BioAssay Systems (catalog EALT-100), as instructed by the kit manufacturers. The blood glucose measurements were performed via tail snips using the Contour Next blood glucose monitoring system from Ascensia Diabetes Care. Mouse-specific test strips were provided by the same company (Countour Next test strips, catalog 84191451).

### Hepatic triglyceride content.

Pieces of central liver lobe (100 mg) were used for hepatic lipid extraction via homogenization in hexane/isopropanol (3:2) using a Tissue Lyzer II (Qiagen). The homogenate was agitated in a rotator for 2 hours at 24°C and then centrifuged (at 14000 x g) to recover the liquid phase. In the supernatant, Na_2_SO_4_ was added to separate the organic from aqueous phase. The organic lipid-containing phase was transferred into a new tube and evaporated overnight under a fume hood. The lipids were dissolved in a Triton X-100/methanol/butanol (1:1:3) solution. The triglyceride measurements were performed as instructed from kit manufacturer (Roche, 11877771). Hepatic triglyceride content was calculated in mg/g liver tissue.

### Histological analysis and stainings.

Histological analysis was conducted as described previously (43, 77, 78). Briefly, for paraffin sectioning, livers were dissected, fixed in 4% paraformaldehyde, and embedded in paraffin. For liver cryosections, freshly isolated liver tissues were embedded in OCT embedding medium without fixation, and samples were frozen in liquid nitrogen. Paraffin-embedded tissue sections (5 μM thick) were used for H&E staining or for staining with 0.1% Sirius red (MilliporeSigma) dissolved in saturated picric acid. Accumulation of ROS was measured by staining of frozen liver sections (5 μm thick) with 2 mM of 5-(and-6)-chloromethyl-20,70-dichlorodihydrofluorescein diacetate, acetyl ester (CM-H_2_DCFDA) for 30 minutes at 37°C, as previously described (43, 77, 78). For Oil Red O staining, HepG2 cells or liver cryosections (10 μm) were fixed in a 4% formaldehyde/PBS at room temperature for 15 minutes and stained with 0.2% Oil Red O stain (MilliporeSigma) for 30 minutes at room temperature to examine intracellular lipid accumulation. Sections were counterstained with hematoxylin, and slides were observed in a Zeiss Apotome 2 microscope.

### Liver IHC.

For liver immunostainings, cryosections were used as described previously (43, 77). Cryosections (5 μm thick) were air dried and fixed with 4% formaldehyde for 15 minutes at room temperature. Blocking was performed in 1% BSA/0,1% Triton X-100 for 1 hour, and sections were incubated with the desired primary antibodies at room temperature for 1 hour or at 4°C overnight: F4/80 (eBioscience, 1:100, 14-4801-82), HNF4α (Santa Cruz Biotechnology Inc., 1:100, SC8987), αSMA (Abcam, 1:100, 5694), and TRIM21 (Santa Cruz Biotechnology Inc., 1:100, 25351). Sections were then incubated with Alexa Fluor 568 or 488 secondary antibodies for 1 hour at room temperature; Alexa Fluor 568 goat anti-rabbit (A11011), Alexa Fluor 488 goat anti-rat (A11006), and Alexa Fluor 568 goat anti-mouse (A11004) were used in this study. Sections were finally counterstained with DAPI, and slides were observed using a Zeiss Apotome 2 microscope.

### RNA extraction and qPCR analysis.

Total RNA from liver pieces or cells was prepared through homogenization in Trizol reagent (MilliporeSigma) using a Tissue Lyser II (Qiagen). To each sample, 0.2 volumes of chloroform were added and centrifuged for 15 minutes at 12,000*g* and 4°C. The upper aqueous phase was precipitated with isopropanol for 30 minutes at –20°C. After resuspension in water, RNA samples were digested with 10 units of DNase I (Roche) with RNAse inhibitors (NEB) for 15 minutes at 37°C and repurified with phenol/chloroform extraction following ethanol precipitation. For first-strand cDNA synthesis, 1 μg of total RNA was used in High-Capacity cDNA Reverse Transcription Kit (Applied Biosystems, 4368814) according to manufacturer’s instructions. Gene expression was analyzed by quantitative real-time PCR using KAPA SYBR FAST qPCR master mix (KAPA Biosystems, KK4611) on a Roche Light Cycler 480II. The mRNA relative quantification was calculated using the 2^–ΔCT^ method, and all values were normalizing to reference transcripts *36b4* or *Gapdh*. All reactions were performed in triplicate.

### Whole-cell extract preparation.

For whole-cell protein extracts, cells or liver pieces were collected and immediately homogenized in RIPA Buffer (50 mM Tris [pH 7.5], 1% NP40, 0,5% Na-Deoxycholate, 0.5% SDS, 150 mM NaCl, 1 mM EDTA [pH 8.0]), supplemented with protease and phosphatase inhibitors (Roche) using a Bioruptor sonicator or Tissue Lyser II (Qiagen), respectively. Homogenates were incubated at 4°C for 20 minutes with constant agitation, and the extracted proteins were recovered after centrifugation at 14000 x g for 20 minutes at 4°C and were immediately stored at –80°C.

### Western blot analysis and quantification.

Western blotting was performed using standard procedures. Briefly, protein concentration was determined using Bradford Ultra reagent (Expedeon). Equal amounts of protein were heat denaturated in 2× Laemmli buffer for 5 minutes at 95°C. Tissue or cell extracts, or the immunoprecipitated proteins, were resolved by SDS-PAGE and transferred to a nitrocellulose membrane by electroblotting in a wet chamber from Bio-Rad. Membranes were blocked in TBST buffer (20 mM Tris [pH 7.5], 150 mM NaCl, and 0.1% Tween 20) supplemented with 5% nonfat dry milk (AppliChem) or with 5% BSA (MilliporeSigma) for 1 hour at room temperature and then incubated with primary antibodies overnight at 4°C. Secondary antibodies (Calbiochem) were applied in TBST solution for 1 hour at room temperature. Membrane-bound immune complexes were developed using ECL chemiluminescent kit (Thermo Fisher Scientific) and exposed by chemiluminescence detection using a Fujifilm analyzer (LAS-4000). The Western blot results were quantified by ImageJ (NIH).

### IP assays.

The cells from 10 cm plates were scrapped off, precipitated at 800*g* for 5 minutes at 4^o^C, washed twice with ice-cold PBS, and lysed in RIPA buffer supplemented with inhibitors on ice for 30 minutes. Liver pieces were immediately homogenized in the same buffer. The lysates were then cleared by centrifugation at 4°C, precleared with Dynabeads protein G (Invitrogen), and incubated with the indicated primary antibodies overnight at 4°C with agitation. Following 3 washes with RIPA buffer supplemented with 500 mM NaCl, and an extra 3 washes with RIPA buffer, the magnetic Dynabeads were collected and boiled in 2× SDS loading buffer at 95°C prior to loading of immunoprecipitates to the gel and to Western blotting.

### In vivo ubiquitination assay.

The ubiquitination assay was performed following a protocol described previously (39). The cells were plated into a 10 cm dish and, following an overnight recovery, were transfected with plasmids or siRNAs targeting Trim21 (Dharmacon, J-006563-12 or L-006563-00) indicated in figure legends, according to manufacturer instructions. Two days after cells were harvested, they were lysed in SDS RIPA buffer supplemented with protease and N-Ethylmaleimide (10 mM) inhibitors. The lysates were incubated with the desired antibodies as indicated in figure legends following the above-described IP procedure. The Dynabeads containing the immunoprecipitates were boiled in 2× SDS loading buffer at 95°C prior to loading into the gel and subjected to immunoblotting using either Anti-Ub- (Santa Cruz Biotechnology Inc, 8017) or anti-K63Ub-specific (eBioscience, HWA4C4). The same protocol was used for liver tissue samples.

### Proteomics for A1CF interactome in liver.

Livers from 4 mice fed a ND were lysed, and the endogenous A1CF was immunoprecipitated as described above. For nonspecific binding, IgG was used in IP experiments. The IPs were analyzed by label-free liquid chromatography–tandem mass spectrometry (LC-MS/MS) shotgun proteomics at the Functional Genomics Center Zurich (FGCZ). All MS samples were analyzed using Mascot (Matrix Science; version2.5.1.3), and the results were processed and analyzed with Scaffold (version Scaffold_4.11.0) proteome software. Scaffold was used to validate MS/MS-based peptide and protein identifications with the following criteria. Peptide identifications were accepted if they could be established at greater than 51.0% probability to achieve an FDR less than 0.1% by the Scaffold Local FDR algorithm. Protein identifications were accepted if they could establish the stringent setting of 1% protein FDR and a minimum of 2 peptides per protein with 0.1% peptide FDR. Statistical analysis was performed using 2-tailed *t* test with significance *P* < 0.05, and Benjamini-Hochberg multiple-test correction (*P* < 0.0006) (Supplemental Table 1). Normalized total ion current (TIC) was used as a spectrum quantitative method for volcano plotting.

### Statistics.

Data are expressed as mean ± SD. Each dot in the bar plots or lane in Western blots represents a biological replicate. Statistical significance (thresholds: *P < 0.05; **P < 0.01; ***P < 0.001; ****P < 0.0001) between 2 groups was determined by unpaired 2-tailed Student’s *t* test with Welch’s correction. For multiple-group comparisons, either 1-way or 2-way ANOVA with Sidak’s post hoc test was used as indicated in the figure legends. Animals were sex and age matched. Animal studies were performed without blinding of the investigator. All statistical tests were performed with either GraphPad Prism 9.0 (GraphPad Software) or Microsoft Excel.

### Study approval.

All ethical regulations complied with animal experiments and were approved by the Kantonale Veterinäramt Zürich. Analysis of human liver biopsies was carried out in accordance with the Code of Ethics of the World Medical Association (Declaration of Helsinki 2013, seventh revision) and was approved by the University Hospital Basel ethics committee (EKNZ 2014-362). Written informed consent was obtained from all participants.

### Data availability.

All data are available in this manuscript and in the Supporting Data Values file. Data generated in this study are available from the corresponding author upon request.

## Author contributions

KCN and MS conceived and designed the study. KCN performed most of the experiments. SG performed immunoblots and IHC. MM synthesized the GalNAc-conjugated siRNAs. SW and MHH provided the liver biopsies and clinical data. KCN and MS wrote the manuscript. All authors read and edited the manuscript.

## Supplementary Material

Supplemental data

Supplemental table 1

Supplemental table 2

Supplemental table 3

Supporting data values

## Version 1 11/08/2023

Electronic publication
