# Supplementary material for: Inflammation-induced TRIM21 represses hepatic steatosis by promoting the ubiquitination of lipogenic regulators
Source: JCI Insight. 2023 Nov 8;8(21):e164694. doi: 10.1172/jci.insight.164694 (PMC10721265; doi:10.1172/jci.insight.164694)
Supplement: Supplemental data [file jciinsight-8-164694-s293.pdf]

Supplemental Figure 1

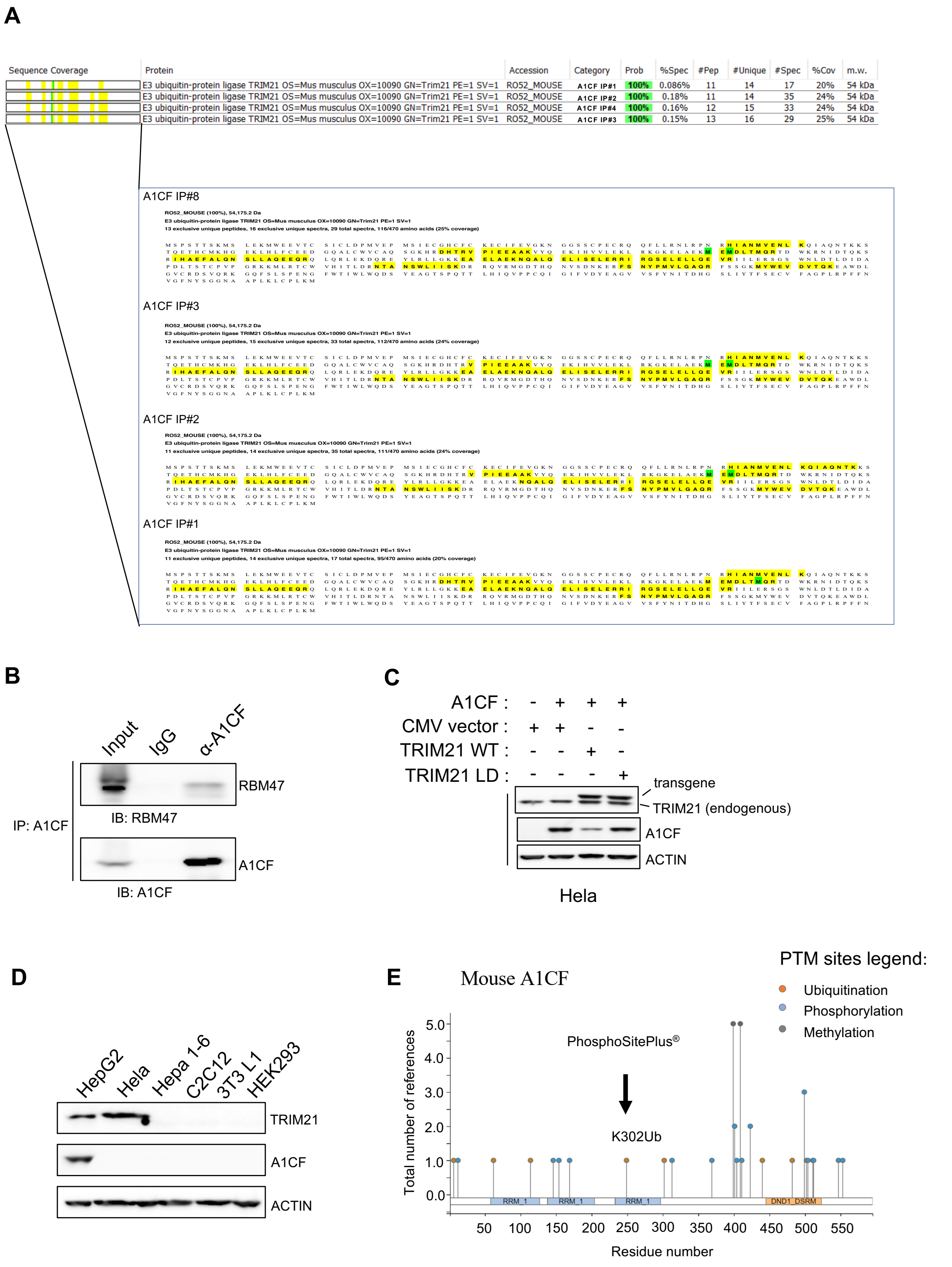

Supplemental Figure 1

Identification and characterization of of A1CF interacting proteins.

A) Whole cell extracts from four adult mouse livers were subjected to immunoprecipitation with anti-A1CF and analyzed by labelled free LC-MS/MS shotgun proteomics to detect the interaction partners of A1CF in liver. The percent of protein coverage (20–25%) and sequence covered from the identified peptides is shown in yellow. Protein probability, number of unique peptides and total spectrum counts were analyzed by Scaffold.

(B) Co-IP assay was performed to validate the endogenous interaction between A1CF and RBM47 in liver extracts of adult C57BL/6 mice.

(C) Hela cells were co-transfected with A1CF and either TRIM21 wildtype (WT), or its ligase-dead (LD) form, and A1CF expression was measured by immunoblotting. Empty CMV vector is used as a control.

(D) A1CF and TRIM21 protein expression of the indicated cell lines. β-ACTIN is used as a loading control.

(E) Schematic illustration of currently identified posttranslational modifications in A1CF. Data assembled from www.phosphosite.org.

Supplemental Figure 2

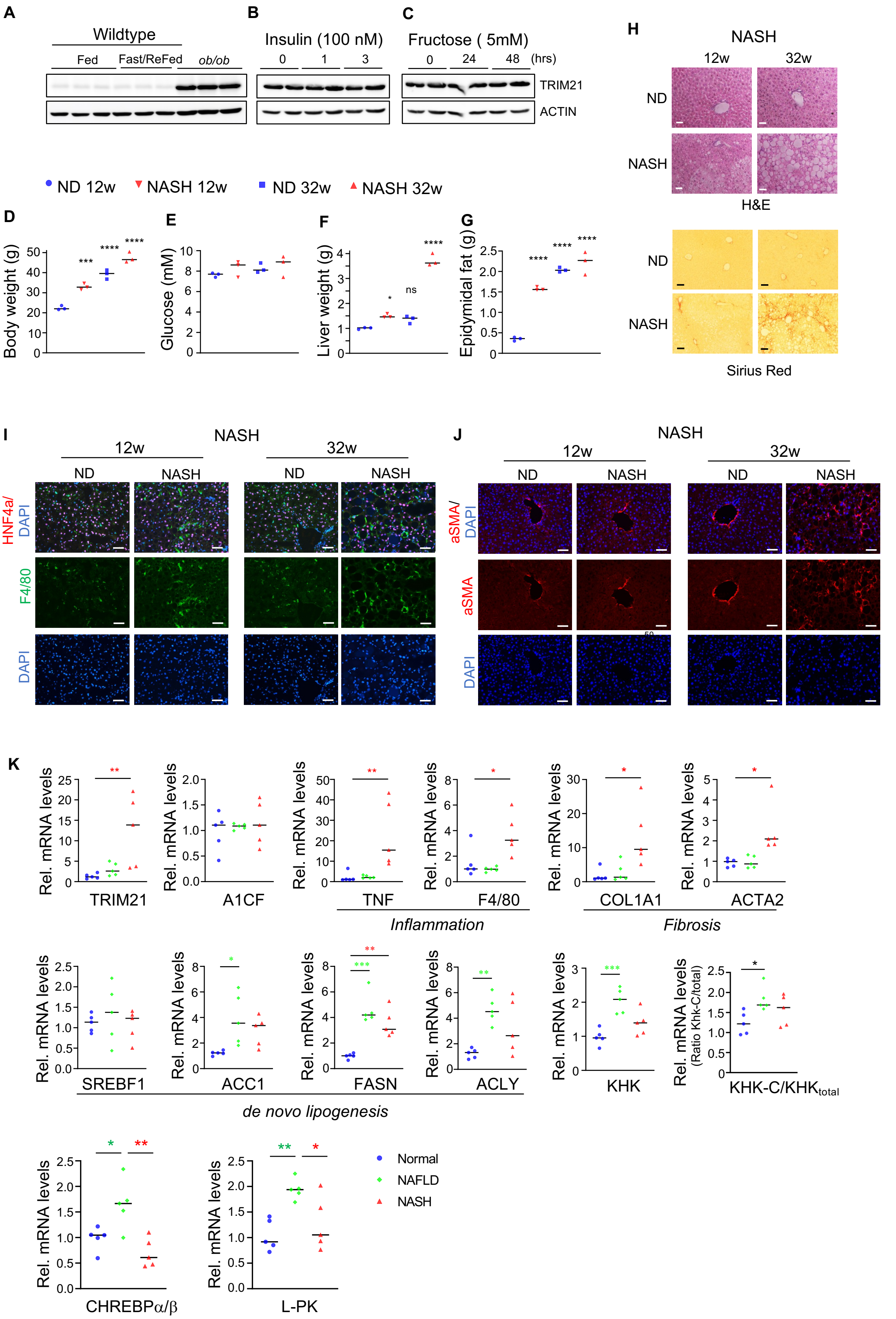

Supplemental Figure 2

Expression of TRIM21 in physiological and metabolic stress conditions

(**A–C**) Immunoblot analysis of TRIM21 expression in livers of chow fed, overnight fasted and refed mice for 2h, and obese mice (*ob/ob*) (**A**), n=3 each group, or in HepG2 cells stimulated with 100 nM insulin (**B**), or in 5 mM fructose (**C**) for the indicated timepoints (n=2). ACTIN is used as a loading control.

(**D–G**) Body weight (BW)(**D**), plasma blood glucose (BG)(**E**), liver (**F**) and epidydimal fat weight (**G**) measurements of mice fed a normal or NASH diet for 12 and 32 weeks (w), n=3 mice per group.

(**H**) Histological analysis of livers from mice indicated as in (**D**–**G**). Bar sizes: H&E: 50 μm, Sirius-Red: 100 μm.

(**I** and **J**) Representative images of staining for F4/80 and HNF4a (**I**) and a-SMA (**J**) in liver sections of mice indicated as in **D**–**G**. Bar size: 50 μm.

(**K**) Relative mRNA expression of indicated genes in livers of healthy individuals without steatosis, simple steatosis (NAFLD) or NASH. The KHK splicing analysis is calculated as the ratio of KHK-C isoform expression in relation to total *KHK* in liver biopsies of healthy, NAFLD and NASH individuals (n=5 mice per group).

In all statistical plots, data are expressed as mean ± SD; ****p<0.0001; ***p<0.001; **p<0.01; *p<0.05; ns: not significant. For **D**–**G** and K the statistical analysis was carried out by one-way ANOVA with Sidak's post-hoc analysis.

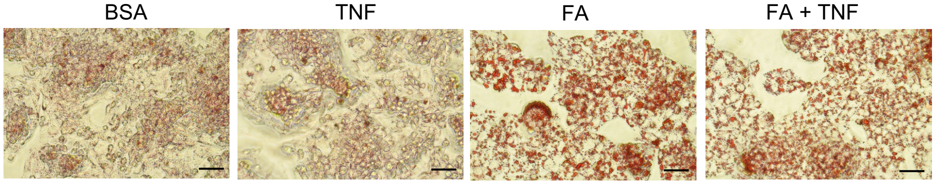

Supplemental Figure 3

Intracellular triglyceride levels in liver cells stimulated with under inflammatory/ metabolic stress conditions

Oil Red O staining in HepG2 cells stimulated with TNF (20 ng/ml), fatty acids (FA) (0.5 mM mixture of oleic and palmitic acid) or combination of TNF and FA for 48 h. Bar size: 50 µm.

Supplemental Figure 4

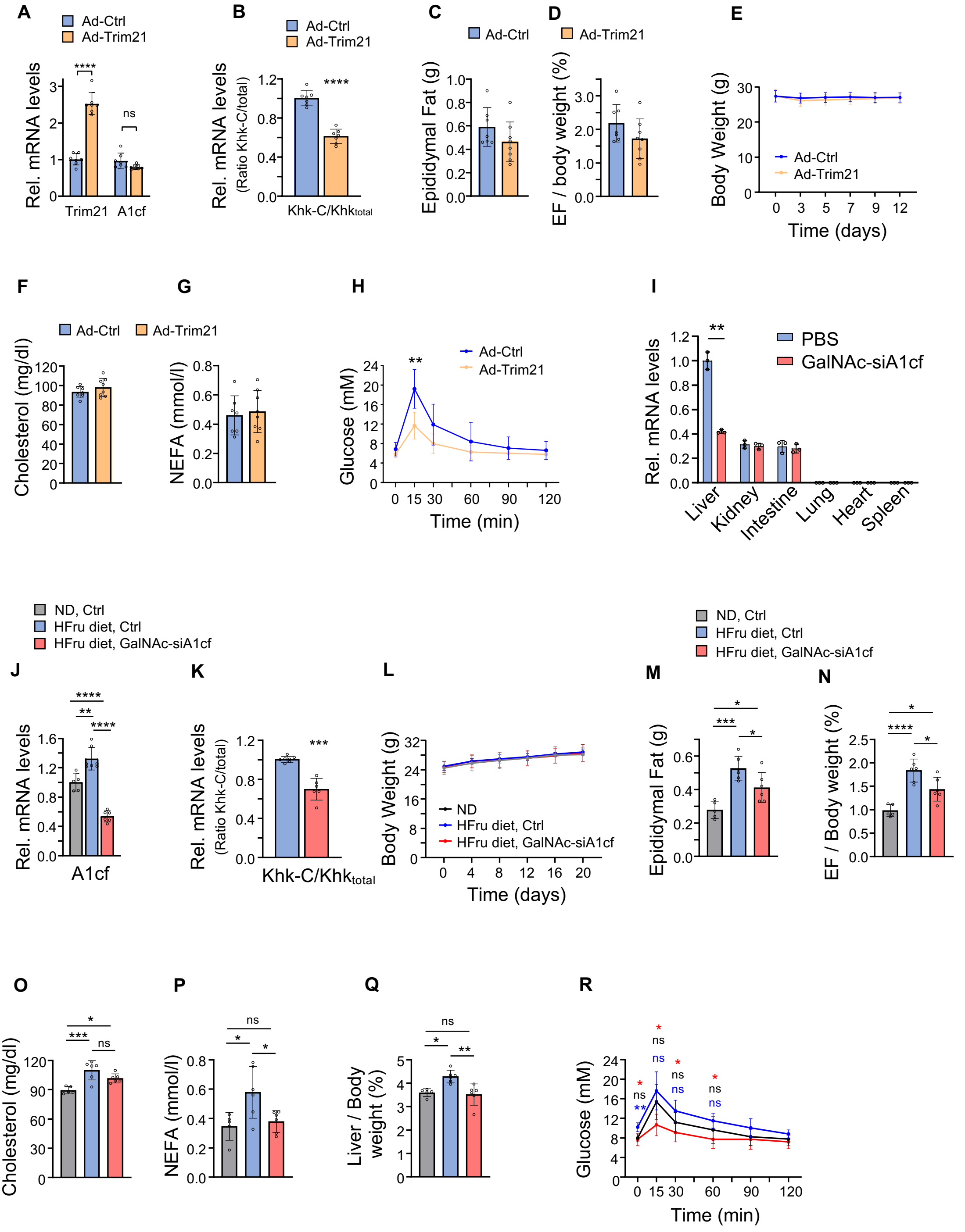

Supplemental Figure 4

Metabolic effects of hepatic Trim21 overexpression or silencing

(A and **B)** Relative mRNA expression of Trim21 and A1cf (**A**), and ratios of Khk-C isoform expression in relation to Khk total levels (**B**) in Ad-Trim21 and Ad-Ctrl injected livers of 12-weeks old mice fed HFru diet for 4 weeks, n=7 mice/group.

(C and D) Epididymal fat (EF)(**C**), and ratios of epididymal fat to body weight (**D**) from mice indicated as in (**A** and **B)**.

(E) Body weight measurements of mice, as described in (**A** and **B)**.

(F and **G)** Plasma cholesterol (**F**) and non-esterified fatty acids (NEFA) levels (**G**) from mice as described in (**A** and **B)**.

(H) Glucose tolerance test (2 g/kg Glu) of mice as indicated in (**A** and **B)**.

(I) Relative A1cf mRNA expression at the indicated tissues from GalNAcsiA1cf and control injected 8-week-old mice, n=3 per group.

(J) Relative A1cf expression levels in liver of C57BL/6 mice fed a chow diet (ND), or high fructose diet and either injected with a control siRNA or GalNAc-siA1cf.

(K) Ratio of Khk-C isoform expression in relation to Khk total levels in livers of GalNAc-siA1cf injected or control C57BL/6 mice fed a HFru diet for 4 weeks. Mice/group: HFru controls (n=7) or GalNAc-siA1cf (n=6).

(L–R) Body weight (**L**), epidymidal fat (EF)(**M**) and ratios of EF to body weight **(N),** plasma cholesterol (**O**) and NEFA (**P**) levels, liver weight to body weight ratio **(Q)**, and glucose tolerance test (2 g/kg Glu) **(R)** of mice indicated as in **J**. Asterisks in blue=ND vs HFru diet: Controls; black: ND vs HFru diet: GalNAc-siA1cf; red: HFru diet groups: controls vs GalNAc-siA1cf.

Mice per group for **C–H**: Ad-Ctrl (n=7), Ad-Trim21 (n=8); for **J, L–R**: ND (n=5), and controls or GalNAc-siA1cf of HFru group (n=6 each). In all statistical plots, data are expressed as mean ± SD; ****p<0.0001; ***p<0.001; **p<0.01; *p<0.05; ns: not significant. Statistical analysis for **A–D, F, G, I** and **K** was carried out by t-test, for **E, H, L and R** by two-way ANOVA with Sidak’s post-hoc analysis; for **J, M–Q** by one-way ANOVA with Sidak’s post analysis.

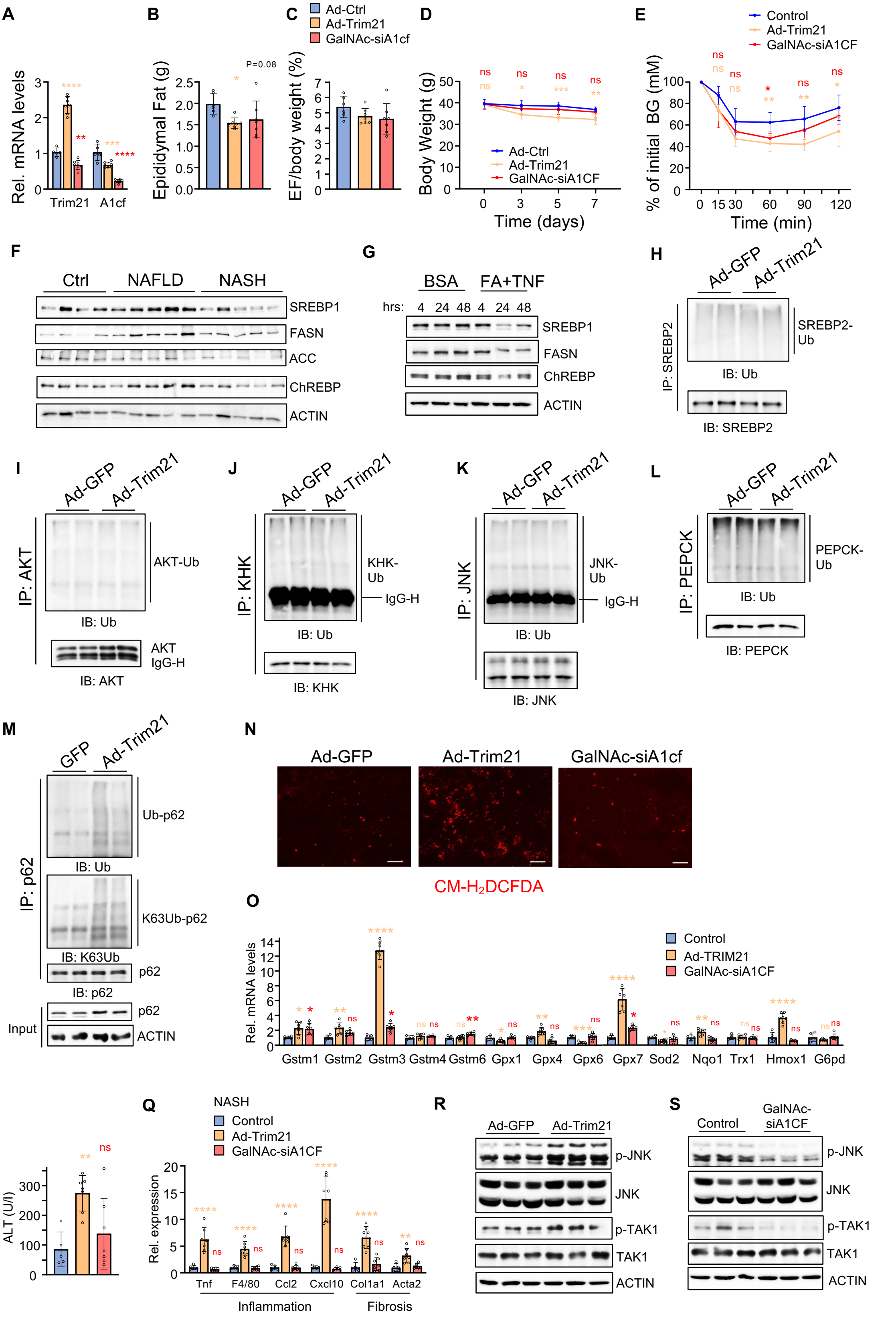
A
Rel. mRNA levels
Trim21
A1cf
B
Epididymal Fat (g)
P=0.08
C
Ad-Ctrl
Ad-Trim21
GalNAc-siA1cf
EF/body weight (%)
D
Body Weight (g)
Ad-Ctrl
Ad-Trim21
GalNAc-siA1CF
Time (days)
E
Control
Ad-Trim21
GalNAc-siA1CF
% of initial BG (mM)
Time (min)
F
Ctrl
NAFLD
NASH
SREBP1
FASN
ACC
ChREBP
ACTIN
G
BSA
FA+TNF
hrs: 4 24 48 4 24 48
SREBP1
FASN
ChREBP
ACTIN
H
Ad-GFP
Ad-Trim21
IP: SREBP2
SREBP2-Ub
IB: Ub
IB: SREBP2
I
Ad-GFP
Ad-Trim21
IP: AKT
AKT-Ub
IB: Ub
AKT
IgG-H
IB: AKT
J
Ad-GFP
Ad-Trim21
IP: KHK
KHK-Ub
IgG-H
IB: Ub
IB: KHK
K
Ad-GFP
Ad-Trim21
IP: JNK
JNK-Ub
IgG-H
IB: Ub
IB: JNK
L
Ad-GFP
Ad-Trim21
IP: PEPCK
PEPCK-Ub
IB: Ub
IB: PEPCK
M
GFP
Ad-Trim21
IP: p62
Ub-p62
IB: Ub
K63Ub-p62
IB: K63Ub
p62
IB: p62
Input
p62
ACTIN
N
Ad-GFP
Ad-Trim21
GalNAc-siA1cf
CM-H2DCFDA
O
Rel. mRNA levels
Control
Ad-TRIM21
GalNAc-siA1CF
Gstm1 Gstm2 Gstm3 Gstm4 Gstm6 Gpx1 Gpx4 Gpx6 Gpx7 Sod2 Nqo1 Trx1 Hmox1 G6pd
P
ALT (U/l)
Q
NASH
Control
Ad-Trim21
GalNAc-siA1CF
Rel. expression
Tnf F4/80 Ccl2 Cxcl10 Col1a1 Acta2
Inflammation
Fibrosis
R
Ad-GFP
Ad-Trim21
p-JNK
JNK
p-TAK1
TAK1
ACTIN
S
Control
GalNAc-siA1CF
p-JNK
JNK
p-TAK1
TAK1
ACTIN

Supplemental Figure 5

Effects of hepatic Trim21 overexpression or A1CF silencing on metabolism and inflammation

(A) Relative mRNA expression of Trim21 and A1cf in livers of Ad-Trim21 injected, GalNAc-siA1cf and control injected mice fed a NASH diet for 20 weeks.

(B–D) Epididymal fat (EF)(**B**) and ratios of EF to body weight (**C**), total body weight (**D**) and insulin tolerance test of mice indicated as in (**A**), controls (n=6), Ad-Trim21 or GalNAc-siA1cf (n=7 each). Statistics in (**D** and **E**): red: Ctrl vs GalNAc-siA1cf; orange: Ctrl vs Ad-Trim21,

(F) Immunoblot analysis of indicated proteins from biopsies of healthy (Ctrl)(n=4), NAFLD or NASH patients (n=5 each).

(G) Immunoblot analysis of indicated proteins from HepG2 cells stimulated with FA (0.5 mM mixture of oleic and palmitic acid) and TNF (20 ng/ml) for the indicated time points.

(H–L), Endogenous ubiquitination assays of SREBP2 (**H**), AKT1/2 (**I**), KHK (**J**), JNK (**K**) and PEPCK (**L**) in livers of Ad-Trim21 and Ad-Ctrl injected mice (n=2 mice per group).

(M) Endogenous total and K63-linked ubiquitination of p62 from livers of mice injected with control (Ad-GFP) or Ad-Trim21. Each lane represents one animal.

(N) Immunostaining using the H_2O_2-sensitive fluorescence dye CM-H2DCFDA in livers of mice fed a NASH diet for 20 weeks and injected as indicated in (**A**). Bar size: 100 µm.

(O) Expression levels of antioxidant genes in livers of NASH mice indicated as in (**A**).

(P) Plasma ALT levels of mice indicated as in (**A**), controls (n=5), Ad-Trim21 or GalNAc-siA1cf (n=7 each)

(Q) Relative mRNA expression levels of indicated inflammation and fibrosis markers in livers of mice indicated as in (**A**).

(R and S) Western blot analysis of indicated proteins in livers of mice injected with Ad-Trim21 (**R**) or GalNAc-siA1CF and controls (**S**), respectively. Each lane represents an individual animal.

Mice/group for **A**, **O** and **Q**: Controls (n=5), Ad-Trim21 or GalNAc-siA1cf (n=7 each). In all statistical plots, data are expressed as mean ± SD; ****p<0.0001; ***p<0.001; **p<0.01; *p<0.05; ns: not significant. Statistical analysis for **A–C, N, O** and **P** was carried out by one-way and for **C** and **D** by two-way ANOVA with Sidak's post hoc analysis.

Supplemental Figure 6

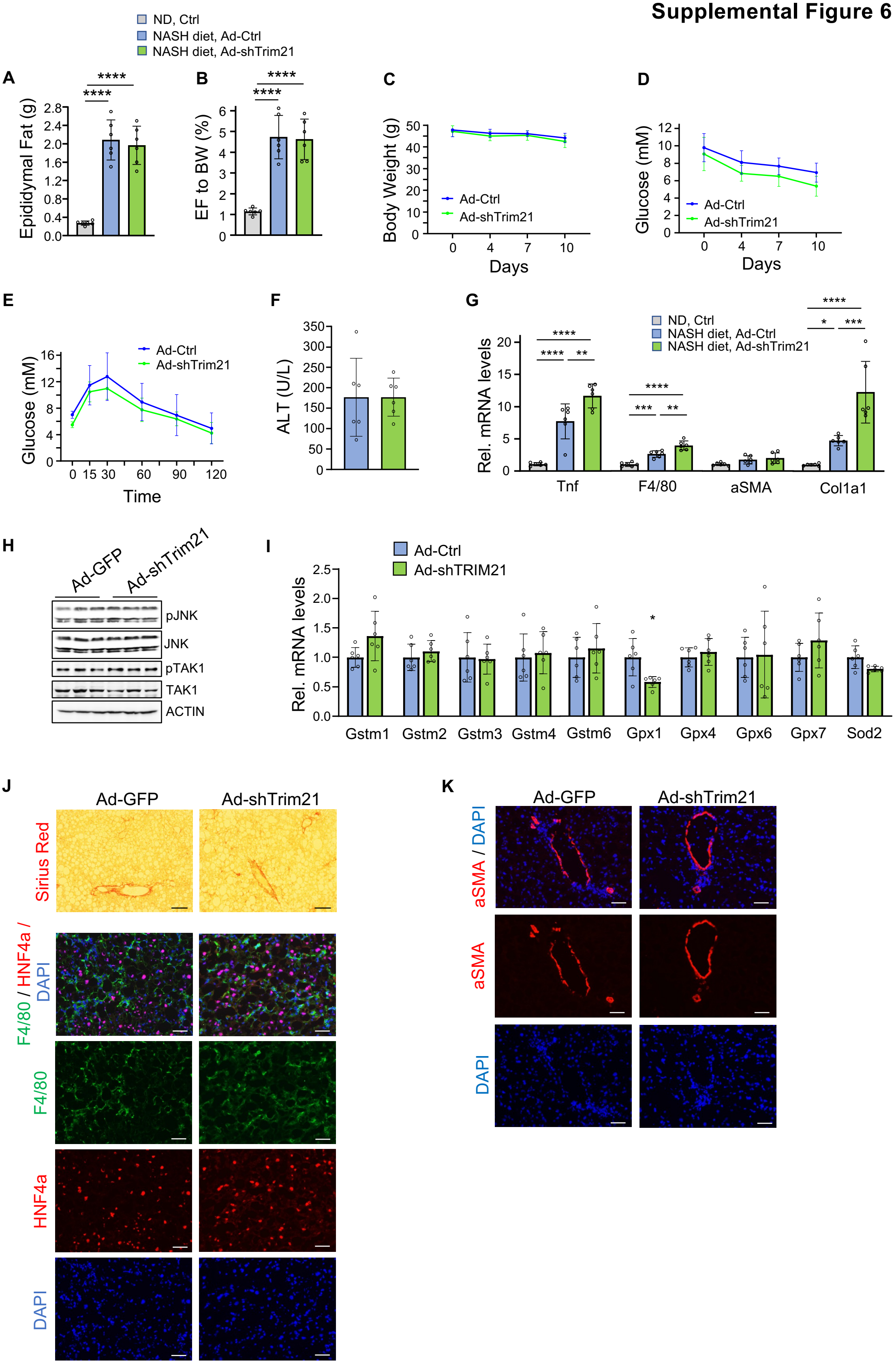

Supplemental Figure 6

Effect of hepatic Trim21 silencing in NASH model

(A and **B)** Epidydimal fat (EF)(**A**) and ratios of EF to body weight (**B**) of C57BL6 mice fed a normal chow diet, or a NASH diet for 30 weeks and either injected with Ad-shTrim21 or Ad-Ctrl adenoviruses.

(C–F) Body weight (**C**) blood glucose (**D**), fructose tolerance test (FTT) (**E**), and plasma ALT (**F**) from C57BL6 mice fed a NASH diet for 30 weeks and injected with Ad-shTrim21 or Ad-Ctrl viruses.

(G) Relative mRNA expression of inflammation and fibrosis markers in livers of mice indicated as in (**A**) and (**B**).

(H) Western blot analysis of the indicated proteins in livers of mice indicated as in (**C**–**F**).

(I) Relative mRNA expression of antioxidant genes in livers of mice indicated as in (**C**–**F**).

(J) Sirius Red and immunostainings with the indicated antibodies in livers of mice indicated as in (**B**). Bar sizes: Sirius Red: 100 µm, immunostainings: 50 µm.

For (**A**–**G**) and (I)**,** n=6 mice per group. In all statistical plots, the data are expressed as mean ± SD; ****$p<0.0001$; ***$p<0.001$; **$p<0.01$; *$p<0.05$; ns: not significant. Statistical analysis for (**A**) and (**G**) was carried out by one-way ANOVA and for (**C**–**E)** by two-way ANOVA with Sidak's post hoc analysis, and for (**F**) and (**I**) by t-test.
